# Profile of common prostate cancer risk variants in an unscreened Romanian population

**DOI:** 10.1111/jcmm.13433

**Published:** 2017-12-20

**Authors:** Paul D. Iordache, Dana Mates, Bjarni Gunnarsson, Hannes P. Eggertsson, Patrick Sulem, Júlíus Guðmundsson, Stefania Benónísdóttir, Irma Eva Csiki, Stefan Rascu, Daniel Radavoi, Radu Ursu, Catalin Staicu, Violeta Calota, Angelica Voinoiu, Mariana Jinga, Gabriel Rosoga, Razvan Danau, Sorin Cristian Sima, Daniel Badescu, Nicoleta Suciu, Viorica Radoi, Andrei Manolescu, Thorunn Rafnar, Bjarni V. Halldórsson, Viorel Jinga, Kári Stefánsson

**Affiliations:** ^1^ School of Science and Engineering Reykjavik University Reykjavik Iceland; ^2^ National Institute of Public Health Bucharest Romania; ^3^ deCODE genetics/AMGEN Reykjavik Iceland; ^4^ Urology Department ‘Prof. Dr. Th. Burghele’ Clinical Hospital University of Medicine and Pharmacy “Carol Davila” Bucharest Romania; ^5^ Department of Medical Genetics Faculty of Medicine “Carol Davila” University of Medicine and Pharmacy Bucharest Romania; ^6^ Carol Davila University of Medicine and Pharmacy Dr. Carol Davila Central University Emergency Military Hospital Bucharest Romania; ^7^ Faculty of Medicine School of Health Sciences University of Iceland Reykjavik Iceland

**Keywords:** prostate cancer, GWAS, Romania, genome‐wide association study, unscreened population

## Abstract

To find sequence variants affecting prostate cancer (PCA) susceptibility in an unscreened Romanian population we use a genome‐wide association study (GWAS). The study population included 990 unrelated pathologically confirmed PCA cases and 1034 male controls. DNA was genotyped using Illumina SNP arrays, and 24.295.558 variants were imputed using the 1000 Genomes data set. An association test was performed between the imputed markers and PCA. A systematic literature review for variants associated with PCA risk identified 115 unique variants that were tested in the Romanian sample set. Thirty of the previously reported SNPs replicated (*P*‐value < 0.05), with the strongest associations observed at: 8q24.21, 11q13.3, 6q25.3, 5p15.33, 22q13.2, 17q12 and 3q13.2. The replicated variants showing the most significant association in Romania are rs1016343 at 8q24.21 (*P* = 2.2 × 10^−4^), rs7929962 at 11q13.3 (*P* = 2.7 × 10^−4^) and rs9364554 at 6q25.2 (*P* = 4.7 × 10^−4^). None of the variants tested in the Romanian GWAS reached genome‐wide significance (*P*‐value <5 × 10^−8^) but 807 markers had *P*‐values <1 × 10^−4^. Here, we report the results of the first GWAS of PCA performed in a Romanian population. Our study provides evidence that a substantial fraction of previously validated PCA variants associate with risk in this unscreened Romanian population.

## Introduction

Prostate cancer is the fourth most common cancer and the second most common cancer in men worldwide 1. Prostate cancer is the third most commonly diagnosed cancer in Europe and has emerged as the most frequent cancer in men, reaching an age‐standardized rate of 96 per 100,000 men in 2012 2. Incidence has been increasing rapidly over the past two decades in most European countries, particularly in the wealthiest countries in Northern and Western Europe 2, 3. More than 1.1 million new cases of prostate cancer were diagnosed in 2012 worldwide, accounting for approximately 8% of all new cancer cases. The incidence is expected to grow to 1.7 million new cases and 500,000 deaths by 2030 worldwide, mainly due to the growth and ageing of the global population 4.

Incidence of prostate cancer differs between countries, in part due to differences in the prevalence of prostate‐specific antigen (PSA) screening. PSA screening has a much greater effect on incidence than on mortality; hence, there is less variation in mortality rates worldwide (10‐fold) than is observed for incidence (25‐fold). In 2012, the age‐standardized mortality rate in Europe was 19 per 100,000 men, and the mortality rate was almost the same in developed and developing regions of Europe 2, 4. Prostate cancer screening with PSA has been shown to decrease prostate cancer mortality in the European Randomized Study of Screening for Prostate Cancer (ERSPC) 5. However, the possibility of negative effects of screening on over‐diagnosis and over‐treatment cannot be ignored 6. Screen‐detected prostate cancer typically runs an indolent course, less than 13% of those diagnosed will succumb to the disease 5. To improve the outcome of screening, it is important to find prognostic biomarkers that can distinguish between indolent and aggressive disease 7. Sequence variants that associate with aggressive PCA could be useful for this purpose.

Genome‐wide association study has been remarkably successful in identifying common sequence variants affecting risk of PCA 8. More than 200 SNPS have been identified at 70 loci, explaining 30% of the familial risk of this disease 9. Most GWAS has been conducted in populations with high rates of PSA screening and includes indolent disease with undetermined clinical significance. Not surprisingly, some of the PCA variants reported have subsequently been shown to associate with PSA levels rather than PCA 10.

In Romania, the estimated age‐standardized incidence of PCA was 37.9 per 100,000 men in 2012, and the estimated age‐standardized mortality rate for PCA was 16.9 per 100,000 men 2. Due to the poor health status of the Romanian population and difficulties in healthcare accessibility 11, PCA might be an underdiagnosed condition. PSA screening is not common in Romania 12 and consequently more than 95% of patients have an advanced disease at the time of diagnosis 13. Here, we report the first GWAS on PCA in Romania and profile the known PCA risk variants in this populations of patients with clinically significant disease.

## Materials and methods

### Study population

Subjects included in this study were male patients admitted between 2008 and 2012 to two clinics in Bucharest (Urology Clinic ‘Th. Burghele’ and General Surgery Clinic ‘St. Mary’) for various medical conditions. The study consists of 2024 hospital patients; 990 unrelated histopathologically confirmed PCA cases, most of which had abnormal PSA levels, and 1034 controls, consisting of patients admitted for urological and surgical conditions other than cancer. Blood samples were collected for the measurement of biomarkers and genotyping. PSA levels in plasma were measured for all subjects at hospital admission but were not used as an exclusion criteria. All subjects gave written informed consent prior to enrolment and accepted the use of personal and clinical data and biological samples for genetic research. The Bioethical Committee of the Romanian College of Physicians approved the study and the study protocols were approved by the National Ethical Board of the Romanian Medical Doctors Association in Romania. Trained interviewers performed face‐to‐face interviews, using standardized questionnaires, to collect personal data (ethnicity, marital status, education, height and weight), lifestyle data (occupation, smoking, coffee and tea consumption) and medical history (personal and familial). All subjects were of self‐reported European descent. No significant difference was observed between the average age of the cases (66.9) and controls (64.3). No significant differences were observed in other epidemiological features: BMI, smoking or alcohol consumptions (Table 1).

**Table 1:** Description of the Romanian case‐control population

	% cases (*n* = 990)	% controls (*n* = 1034)
*Age*		
under 50	0.3	15.5
50–60	1	20.1
60–70	35.5	26.8
70–80	44	31.2
80–90	15	6.1
Over 90	0.2	0.1
*T Staging*		
1A	2.3	–
1B	1.1	–
1C	15.6	–
2A	1.7	–
2B	1.9	–
2C	4.0	–
3A	43.8	–
3B	6.06	–
4	23.3	–
*Gleason Score*		
2	0.2	–
3	0.3	–
4	1	–
5	3.3	–
6	13.2	–
7	45.1	–
8	20.3	–
*N Staging*		
N0	21.5	–
N1	3.2	–
Nx	75.3	–
*M Staging*		
M0	22	–
M1	10	–
Mx	68	–
*PSA levels*		
<4	42	–
4.0–9.99	18	–
9.99–19.99	12	–
19.99–49.99	9.5	–
49.99–99.99	6.7	–
>100	9.3	–
NA	1.5	–

	%cases (*n* = 979)	%controls (*n* = 1030)
*Alcohol consumptions*		
No	43.4	45.1
Yes	56.6	54.9

	% cases (*n* = 975)	% controls (*n* = 1022)
*Smoking*		
No	88.1	81.9
Yes	11.9	19.1

	% cases (*n* = 979)	% controls (*n* = 1023)
*BMI*		
Underweight	1.5	0.5
Normal weight	37.5	31.5
Overweight	45.6	47.7
Obese	15.1	20.3

The UICC–TNM staging system was used 14. For the T stage, more than 75 per cent of the cases were graded as T3 or T4. The N and M stages were distributed similarly, and a vast majority were staged as Mx or Nx. For the Gleason score, the majority of cases were graded as Gleason 7 or 8 (45.1% and 20.3%, respectively). A complete description of the clinical characteristics of the cohort is found in Table 1.

### Genotyping and analysis of SNP data

DNA was extracted from whole blood at deCODE Genetics (Reykjavik, Iceland) and genotyped using Infinium OmniExpress‐24 bead chips (Illumina). A total of 716,503 SNPs were genotyped for each individual included in the study. The genotype data were filtered using Plink! v1.07 15. Approximately 10% of the SNPs genotyped were removed using a Hardy–Weinberg equilibrium significance threshold of 5 × 10^−6^ and by excluding markers with a minor allele frequency lower than 1%. Prior to the imputation, each chromosome was phased in a single run using SHAPEIT 16. Markers from Phase 3 October 2014 of the 1000 Genomes 17 were imputed into the 2024 chip‐typed individuals using the IMPUTE2 software 18 with a posterior probability of 0.9 as a threshold to call genotypes. The set of genotypes were tested for population heterogeneity using principal component analysis in the ADMIXTURE software 19, and the results were consistent with a homogeneous population.

A total of 24,295,558 markers were generated by imputation for each individual in the study. Quality control for the imputation results was performed by removing markers with minor allele frequency less than 1%, call rate of 0.95 and info of 0.8. In total, 8,506,022 markers met the filtering criteria. An association test was performed between the 8.5 million imputed markers and a phenotype represented by positive biopsy for prostate cancer. The association test was calculated using SNPTEST 20, using a single binary variable as a response; all reported *P*‐values are two‐sided.

### Selection of SNPs for replication of previous findings

A systematic literature review of variants associated with prostate cancer from previous GWAS’ was completed on 4 October 2016 using the NHGRI catalogue of published genome‐wide association studies 21 as a starting point. A search query with ‘prostate cancer’ as a keyword was performed, and the inclusion criteria for selection were as follows: *P*‐values <5 × 10^−8^ and a minor allele frequency above 5%. For each study, the following variables were collected: country and ethnicity of the participants, genotyping method, source of controls and source of replication cohort, and number of cases and controls in both discovery and replication study.

A total of 37 articles were originally obtained from the GWAS catalogue based on the keyword search. Twelve of the studies reported results only tangentially related to prostate cancer, while the remaining 25 studies reported associations with prostate cancer risk. After removing duplicate markers, we obtained 173 unique markers. Out of the 173, 58 markers did not report either ORs and corresponding 95% CI or the tested allele. These markers were excluded from the study, resulting in a final set of 115 unique variants used in our replication.

## Results

**Figure 1:**
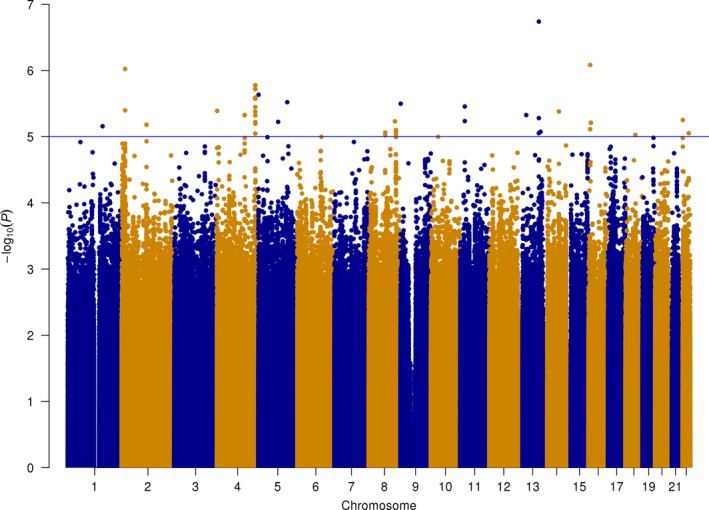
Manhattan plot of GWAS findings in the Romanian sample. Y‐axis shows –log10 *P*‐values and x‐axis shows chromosomal position.

**Figure 2:**
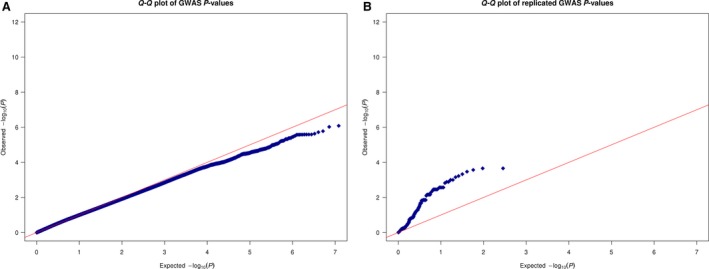
Q‐Q plot of the association results. Blue dots show observed *P*‐values, and red line shows expected *P*‐values. (**A**) Shows results from genome‐wide analysis; (**B**) Shows results when restricted to GWAS catalogue markers.

To search for new susceptibility loci for prostate cancer, we tested a total of 8.5 million variants of frequency above 1%. No variants tested in the Romanian GWAS reached genome‐wide significance (*P*‐value lower than 5 × 10^−8^), while 635 markers showed association *P*‐values <1 × 10^−4^ (Supplementary Table 1) and 41 markers, at 16 genetic loci, showed association *P*‐values <1 × 10^−5^. Figure 1 shows a Manhattan plot of the results. The 16 markers with the lowest *P*‐values at each locus are shown in Table 2. We observe no excess signal in the Q‐Q plot when testing all marker (Fig. 2A); the observed *P*‐values (blue line) show a comparable trend to the expected *P*‐values (the red line).

**Table 2:** The variants in the Romanian GWAS with lowest *P*‐values for each locus MAF = Minor Allele Frequency

RS ID	Chromosome	Position	Reference allele	Tested allele	Info	MAF* (%)	OR	*P*‐value
rs55960139	13	95288608	T	C	0.93	22.3	1.45	1.83 × 10^−7^
rs146493482	16	8002169	C	T	0.93	2.4	2.86	8.25 × 10^−7^
rs17467679	2	16133863	A	G	1	37.8	0.74	9.48 × 10^−7^
rs35890542	4	177243229	A	G	1	6.6	0.54	1.67 × 10^−6^
rs187936586	11	21614186	T	C	0.88	2.4	0.38	3.50 × 10^−6^
rs13111983	4	710801	T	G	0.91	27.1	0.74	4.08 × 10^−6^
rs1383	14	73129765	T	A	0.83	23.4	1.36	4.18 × 10^−6^
rs6834053	4	127918594	C	A	0.93	3.9	2.14	4.70 × 10^−6^
rs35544574	13	37172379	CAA	C	0.95	9.2	1.6	4.72 × 10^−6^
rs74437803	22	17089228	G	A	0.81	8.4	0.63	5.60 × 10^−6^
rs71751677	16	11314438	GTGTTT	G	0.86	48.7	0.78	6.16 × 10^−6^
rs201872456	2	115129517	C	G	0.8	17.3	1.38	6.59 × 10^−6^
rs183478269	1	161032417	G	C	0.82	1.5	3.08	6.97 × 10^−6^
rs13253942	8	126154649	G	A	1	9.5	1.63	7.93 × 10^−6^
rs148921321	8	76468497	C	T	0.93	2.1	0,31	8.67 × 10^−6^
rs133917	22	44524314	C	T	0.81	47.3	1.27	8.89 × 10^−6^

Next, we tested the effect of 115 previously reported PCA variants in the Romanian population. Thirty SNPs from 13 loci replicated in the Romanian cohort (*P*‐value <0.05) (Table 3). Eighty‐nine (77%) of the markers selected in the systematic literature review show effects consistent with reported studies although the *P* values were not <0.05. We observe an excess of signal in the Q‐Q plot when restricted to this set of previously reported variants (Fig. 2B); the observed *P*‐values (blue line) show a steeper slope than the expected *P*‐values (the red line).

**Table 3:** Previously reported PCa risk markers that associated with PCa risk in the Romanian population with *P* value < 0.05

Rs Number	Chr	Position	OR (95% CI)	*P*‐value	Tested allele	Mapped gene	eQTL genes
rs636291	1	10496040	1.19 (1.05, 1.35)	8 × 10^−3^	A	PEX14	PEX14, PGD, APITD1
rs1218582	1	154861707	1.13 (1.01, 1.26)	4 × 10^−2^	G	KCNN3	KCNN3, PBXIP1
rs7611694	3	113556777	1.14 (1.01, 1.29)	3 × 10^−2^	A	SIDT1	SIDT1, WDR52
rs7679673	4	105140377	1.15 (1.02, 1.29)	2 × 10^−2^	C	TET2	PPA2
rs2242652	5	1279913	1.23 (1.06, 1.42)	5 × 10^−3^	C	TERT	–
rs7725218	5	1282299	1.21 (1.09, 1.36)	7 × 10^−4^	G	TERT	CTD‐2228K2.7
rs9364554	6	160412632	1.28 (1.11, 1.47)	4 × 10^−4^	T	SLC22A3	SLC22A3
rs7758229	6	160419220	1.23 (1.08, 1.40)	1 × 10^−3^	T	SLC22A3	SLC22A3
rs1016343	8	127081052	1.30 (1.13, 1.50)	2 × 10^−4^	T	PCAT2, PRNCR1	–
rs13254738	8	127092098	1.13 (1.01, 1.28)	4 × 10^−2^	C	PCAT2, PRNCR2	–
rs12682344	8	127094539	1.58 (1.13, 2.20)	7 × 10^−3^	G	PCAT2, PRNCR3	–
rs6983561	8	127094635	1.58 (1.13, 2.21)	7 × 10^−3^	C	PCAT2, PRNCR4	–
rs16901979	8	127112671	1.58 (1.13, 2.21)	7 × 10^−3^	A	PCAT2, PRNCR5	–
rs10505483	8	127112950	1.58 (1.13, 2.21)	7 × 10^−3^	T	PCAT2, PRNCR6	–
rs445114	8	127310936	1.23 (1.09, 1.39)	1 × 10^−3^	T	PCAT2, PRNCR7	–
rs6983267	8	127401060	1.16 (1.03, 1.31)	1 × 10^−2^	G	PCAT2, PRNCR8	CASC8
rs1447295	8	127472793	1.35 (1.11, 1.64)	3 × 10^−3^	A	PCAT2, PRNCR9	–
rs4242382	8	127505328	1.33 (1.10, 1.62)	3 × 10^−3^	A	PCAT2, PRNCR10	–
rs4242384	8	127506309	1.33 (1.10, 1.62)	3 × 10^−3^	T	PCAT2, PRNCR11	–
rs10090154	8	127519892	1.32 (1.09, 1.60)	4 × 10^−3^	T	PCAT2, PRNCR12	–
rs11228565	11	69211113	1.19 (1.03, 1.38)	2 × 10^−2^	A	MMP7, MMP20	–
rs7929962	11	69218116	1.25 (1.11, 1.40)	3 × 10^−4^	T	MMP7, MMP20	RP11‐554A11.9
rs7931342	11	69227030	1.23 (1.09, 1.39)	6 × 10^−4^	G	MMP7, MMP20	RP11‐554A11.9
rs10896449	11	69227200	1.24 (1.10, 1.40)	3 × 10^−4^	G	MMP7, MMP20	RP11‐554A11.9
rs11568818	11	102530930	1.19 (1.05, 1.34)	5 × 10^−3^	A	MMP7, MMP20	MMP7
rs10875943	12	49282227	1.16 (1.02, 1.32)	3 × 10^−2^	C	TUBA1C	FKBP11, LMBR1L, TUBA1C, C1QL4, RP11‐386G11.10
rs4430796	17	37738049	1.16 (1.03, 1.31)	2 × 10^−2^	A	HNF1B	–
rs8064454	17	37741595	1.20 (1.06, 1.35)	3 × 10^−3^	C	HNF1B	–
rs2735839	19	50861367	1.20 (1.01, 1.42)	3 × 10^−2^	G	KLK3	–
rs5759167	22	43104206	1.21 (1.08, 1.36)	1 × 10^−3^	G	BIK	–

**Figure 3:**
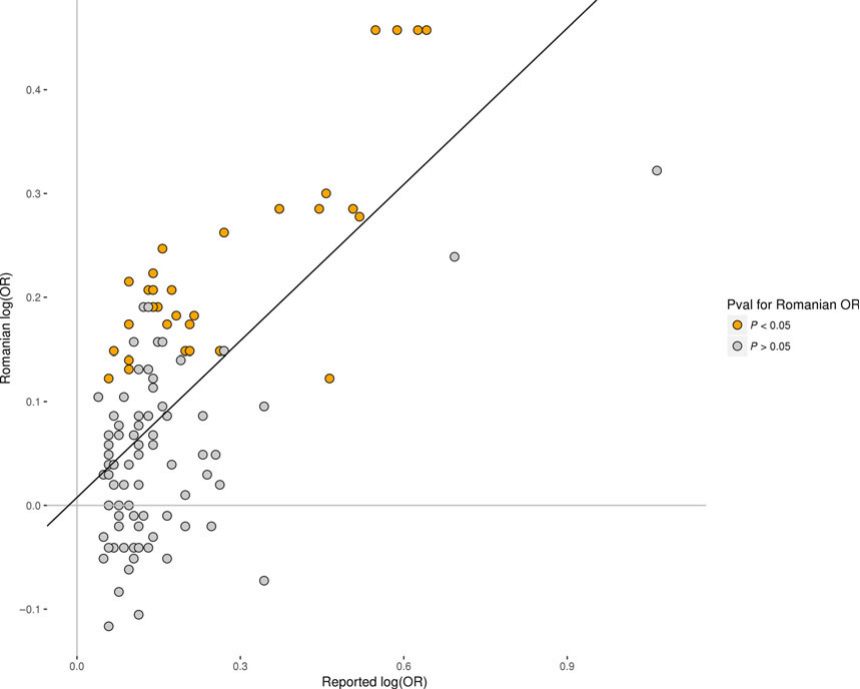
Scatter plot showing the association of the 115 previously reported SNPs with PCA [log(OR)] in the Romanian data set (*x*‐axis) and in reported articles (*y*‐axis).

Replication, or lack thereof, allows us to refine association signals and rule out associations due to differences in phenotype definitions between cohorts. Compared to the original studies, replication studies may use cohorts with slightly different ethnic and pathologic characteristic. Differences in ethnic characteristics lead to differences in LD structure and consequently markers that were previously found to be correlated with a risk variant may not show an association in a population of different ethnicity. We determined whether the effects of the 115 reported SNPs are similar in the Romanian population as in the discovery cohorts, by conducting a weighted linear regression, modelling the relationship between the log‐odds ratio of each of the 115 SNP (Fig. 3). We observed a highly significant correlation of *R* = 0.66 (*P*‐value = 5 × 10^−16^) for the 115 markers represented by the grey (non‐replicating) and orange (replicating) dots. Most markers are near the diagonal, indicating that the effect in the Romanian population is similar to that previously reported.

The locus showing the strongest replication in the Romanian GWAS is 8q24 represented by 12 variants with *P*‐values ranging from 2 × 10^−4^ to 4 × 10^−2^. These 12 SNPs are in high LD (average *R* ^2^ = 0.81) clustering in a 500 kb region, all representing the same association signal. The closest gene to this locus is the *MYC* gene. The locus showing the second strongest replication in Romania is 11q13.3 located close to the *MYEVO* gene 20. This locus is represented by 4 SNPs with *P*‐values between 2.7 × 10^−4^ and 2.1 × 10^−2^. All four SNPs are in high LD (R^2^ >0.93) clustering in a 10KB region and represent the same association signal. This locus was previously reported to associate with early‐onset PCA 22. We assessed the association with early‐onset PCA in the Romanian cohort using the same criteria as in the original study, but could not replicate this result (*P* = 0.41, OR = 0.81), possibly due to lack of power in our set of 128 early‐onset PCA cases. The locus showing the third strongest replicated association in the Romanian results is 6q25.3, represented by a pair of markers (rs7758229 *P* = 1.5 × 10^−3^ and rs9364554 *P* = 4.7 × 10^−4^) in strong LD (*R* ^2^ > 0.78). The markers are located in the proximity of *SLC22A3*, a gene that has been implicated in prostate cancer pathogenesis 23. The 17q12 locus was replicated by a pair of markers in high LD (rs8064454 *P* = 3.1 × 10^−3^ and rs4430796 *P* = 1.5 × 10^−2^, *R* ^2^ > 0.96) clustering in a 5KB region next to the *HNF1B* gene, representing the same association signal.

## Discussion

Genetic epidemiology straddles between statistically driven research and research inspired by clinical needs. Genome‐wide association studies have successfully yielded loci associated with PCA risk; however, none of the variants at these loci conclusively separate aggressive from indolent disease. Most previous GWAS’ investigating PCA are based on cohorts including indolent cancer forms, including cases with low stage and grade. In an attempt to search for loci of clinical importance, the present study focused on refining associations in men with clinical presentations and not those identified solely by an elevated PSA. More than 70% of the cases included in our study presented with a Gleason score equal to or greater than 7, and a majority were staged at T3 and T4. This is a clear indication of aggressiveness of the tumours; therefore, the replicated variants are likely to represent associations with clinically significant disease although they may also associate with the indolent form of the disease.

At least two studies of similar size have been performed including clinically advanced cases 24, 25. In both studies, the patients had less advanced clinical characteristics than the Romanian cohort. In both studies, fewer than 50% of cases presented with stage T3 and T4 or Gleason score equal to or higher than 7 24, 26. Despite the clinically well‐defined population, no variants tested in the Romanian GWAS reached genome‐wide significance (*P*‐value lower than 5 × 10^−8^). The GWAS Q‐Q plot (Fig. 2A) and the lack of novel genome‐wide significant results suggest that our data set is under‐powered to detect genome‐wide significant associations on its own.

Although only 30 of the 115 previously reported markers showed *P*‐values < 0.05, the effects of additional 59 markers were consistent with the reported results. The ‘winner's curse’, the observation that effect sizes are often larger in the populations in which they are discovered, may be one reason why some SNPs failed to replicate, and why ORs were generally smaller in our cohort than previously found 27. Previous studies have shown the utility of including functional evaluation, in an attempt to identify candidate risk loci below currently accepted statistical levels of genome‐wide significance 28. Functional characterization of the variants described here remains to be done. However, the GTEx database 29 suggests that some of the markers may influence gene expression.

It is interesting to note that many of the variants showing the strongest replication in the Romanian population reside at loci that have been associated with several cancer types, so‐called cancer hubs. The locus showing the strongest replication *P*‐value (2 × 10^−4^) in the Romanian GWAS is 8q24, one of the first hotspots for cancer risk alleles reported. In addition to PCA, the locus was previously reported to associate with breast cancer 30, colorectal cancer 31, 32, ovarian cancer 33, pancreatic cancer 34, renal cell carcinoma 35, urinary bladder cancer 36 and Hodgkin's lymphoma 37. The closest genes to this locus is the *MYC* gene.

A similar situation is found in the case of 11q13.3, a locus associated with breast cancer 38, 39 and early‐onset breast cancer 40, renal cell carcinoma 41 and multiple myeloma 42, in addition to PCA 43, 44 and early‐onset PCA 22.

Yet, another locus replicating in our study that is associated with several types of cancer is the *TERT* locus at 5p15.33. Variants at this locus have been associated with risk of lung cancer 45, pancreatic cancer 46, breast cancer 47, testicular cancer 48 and bladder cancer 49. The two markers replicated in this region, rs2242652 and rs7725218, are both located in the intron region of the *TERT* gene, a gene known to be involved in the activation of oncogenic pathways.

Our study provides evidence that a large fraction of previously validated prostate cancer SNPs associate with risk in the unscreened Romanian population. These variants are likely to have clinical importance and can be considered for inclusion in future risk models of potential clinical utility.

## Conflict of interest

The authors confirm that there are no conflicts of interest.

## Supporting information


**Table S1** Variants with *P*‐values <1 × 10^−4^.
