# Supplementary material for: Profile of common prostate cancer risk variants in an unscreened Romanian population
Source: J Cell Mol Med. 2017 Dec 20;22(3):1574–82. doi: 10.1111/jcmm.13433 (PMC5824401; doi:10.1111/jcmm.13433)
Supplement: Supplementary file 1 — Table S1 Variants with P‐values <1 × 10^−4^. [file JCMM-22-1574-s001.docx]

| RS | chr | pos | A1 | A2 | Info | MAF | OR | P-value |
| --- | --- | --- | --- | --- | --- | --- | --- | --- |
| rs55960139 | 13 | 95288608 | T | C | 0.96 | 0.22 | 1.45 | 1.83E-07 |
| rs146493482 | 16 | 8002169 | C | T | 1.00 | 0.02 | 28.60 | 8.25E-07 |
| rs17467679 | 2 | 16133863 | A | G | 1.00 | 0.37 | 0.74 | 9.48E-07 |
| rs35890542 | 4 | 177243229 | A | G | 1.00 | 0.06 | 0.54 | 1.67E-06 |
| rs72282389 | 4 | 177243796 | CAT | C | 1.00 | 0.06 | 0.54 | 1.91E-06 |
| rs17681035 | 4 | 177244326 | G | A | 1.00 | 0.06 | 0.55 | 2.58E-06 |
| rs34966091 | 4 | 177244388 | G | A | 1.00 | 0.06 | 0.55 | 2.58E-06 |
| rs34773277 | 4 | 177244416 | G | A | 1.00 | 0.06 | 0.55 | 2.58E-06 |
| rs35687562 | 4 | 177244483 | C | T | 1.00 | 0.06 | 0.55 | 2.58E-06 |
| rs79630959 | 4 | 177244970 | T | C | 1.00 | 0.06 | 0.55 | 2.62E-06 |
| rs75407850 | 4 | 177245005 | T | G | 1.00 | 0.06 | 0.55 | 2.62E-06 |
| rs35304696 | 4 | 177245531 | A | T | 1.00 | 0.06 | 0.55 | 2.65E-06 |
| rs71613788 | 4 | 177245672 | A | T | 1.00 | 0.06 | 0.55 | 2.66E-06 |
| rs1454164 | 4 | 177245956 | G | A | 1.00 | 0.06 | 0.55 | 2.68E-06 |
| rs187936586 | 11 | 21614186 | T | C | 0.99 | 0.02 | 0.38 | 3.50E-06 |
| rs141906073 | 4 | 177245578 | TTTG | T | 1.00 | 0.06 | 0.55 | 3.57E-06 |
| rs17467672 | 2 | 16133431 | C | T | 0.99 | 0.36 | 0.75 | 4.01E-06 |
| rs13111983 | 4 | 710801 | T | G | 0.95 | 0.27 | 0.74 | 4.08E-06 |
| rs1383 | 14 | 73129765 | T | A | 0.91 | 0.23 | 13.57 | 4.18E-06 |
| rs36002836 | 4 | 177245843 | A | G | 1.00 | 0.06 | 0.56 | 4.19E-06 |
| rs6834053 | 4 | 127918594 | C | A | 0.99 | 0.03 | 21.36 | 4.70E-06 |
| rs35544574 | 13 | 37172379 | CAA | C | 0.99 | 0.09 | 16.02 | 4.72E-06 |
| rs80078170 | 4 | 127900084 | T | C | 0.99 | 0.03 | 21.36 | 4.76E-06 |
| rs13117427 | 4 | 177255407 | A | G | 1.00 | 0.06 | 0.57 | 4.77E-06 |
| rs9524577 | 13 | 95291254 | T | C | 0.98 | 0.30 | 1.35 | 5.24E-06 |
| rs74437803 | 22 | 17089228 | G | A | 0.96 | 0.08 | 0.63 | 5.60E-06 |
| rs77852312 | 11 | 21603912 | A | G | 0.99 | 0.02 | 0.39 | 5.82E-06 |
| rs35677011 | 4 | 177255048 | C | T | 1.00 | 0.06 | 0.57 | 6.01E-06 |
| rs71751677 | 16 | 11314438 | GTGTTT | G | 0.90 | 0.48 | 0.78 | 6.16E-06 |
| rs35763202 | 4 | 177258250 | G | A | 1.00 | 0.06 | 0.57 | 6.37E-06 |
| rs183478269 | 1 | 161032417 | G | C | 0.99 | 0.01 | 30.80 | 6.97E-06 |
| rs17672470 | 16 | 8021162 | A | G | 1.00 | 0.04 | 19.90 | 7.73E-06 |
| rs13253942 | 8 | 126154649 | G | A | 1.00 | 0.09 | 16.33 | 7.93E-06 |
| rs72651335 | 13 | 103234724 | G | T | 0.99 | 0.06 | 17.58 | 8.43E-06 |
| rs3923300 | 8 | 126142476 | G | C | 1.00 | 0.09 | 16.31 | 8.50E-06 |
| rs148921321 | 8 | 76468497 | C | T | 1.00 | 0.02 | 0.31 | 8.67E-06 |
| rs9524575 | 13 | 95290212 | C | G | 0.97 | 0.30 | 1.34 | 8.86E-06 |
| rs133917 | 22 | 44524314 | C | T | 0.88 | 0.47 | 12.75 | 8.89E-06 |
| rs17062749 | 4 | 177237631 | A | G | 1.00 | 0.08 | 0.60 | 8.98E-06 |
| rs34903473 | 8 | 126156276 | G | C | 1.00 | 0.09 | 16.27 | 9.37E-06 |
| rs118104830 | 8 | 76464176 | G | A | 1.00 | 0.02 | 0.31 | 9.59E-06 |
| rs12211972 | 6 | 111391537 | A | G | 0.94 | 0.33 | 13.05 | 1.01E-05 |
| rs13273034 | 8 | 126134996 | A | G | 1.00 | 0.09 | 16.18 | 1.01E-05 |
| rs56207109 | 5 | 42344171 | C | T | 0.99 | 0.04 | 0.52 | 1.02E-05 |
| rs7259651 | 19 | 51523277 | A | G | 1.00 | 0.28 | 13.41 | 1.04E-05 |
| rs79278292 | 4 | 128110679 | G | A | 0.99 | 0.03 | 22.62 | 1.04E-05 |
| rs5748084 | 22 | 17088114 | A | G | 0.98 | 0.13 | 14.64 | 1.05E-05 |
| rs116269169 | 4 | 128101155 | C | G | 0.99 | 0.03 | 22.62 | 1.05E-05 |
| rs13014858 | 2 | 115192890 | A | G | 1.00 | 0.37 | 0.76 | 1.17E-05 |
| rs74745048 | 4 | 127929760 | T | C | 0.99 | 0.03 | 21.91 | 1.27E-05 |
| rs13004318 | 2 | 3648396 | T | C | 1.00 | 0.43 | 13.03 | 1.28E-05 |
| rs6754510 | 2 | 16141953 | G | C | 0.99 | 0.36 | 0.76 | 1.28E-05 |
| rs3760737 | 19 | 51523807 | G | A | 0.99 | 0.25 | 13.49 | 1.39E-05 |
| rs13067957 | 3 | 141889009 | T | G | 1.00 | 0.09 | 0.62 | 1.41E-05 |
| rs5748093 | 22 | 17088983 | G | T | 0.97 | 0.13 | 14.50 | 1.42E-05 |
| chr17:13174275 | 17 | 13174275 | C | CTTTTTT | 0.98 | 0.24 | 0.74 | 1.42E-05 |
| rs11896089 | 2 | 16171139 | T | A | 0.87 | 0.49 | 12.60 | 1.45E-05 |
| rs13136122 | 4 | 704602 | A | G | 0.99 | 0.25 | 0.74 | 1.48E-05 |
| rs6706154 | 2 | 8398487 | T | C | 1.00 | 0.06 | 17.13 | 1.50E-05 |
| rs58498411 | 2 | 16143245 | A | AT | 0.99 | 0.36 | 0.77 | 1.58E-05 |
| rs6855508 | 4 | 127689118 | T | C | 0.99 | 0.03 | 22.23 | 1.61E-05 |
| rs17055011 | 6 | 108056510 | T | C | 1.00 | 0.02 | 0.37 | 1.64E-05 |
| rs72933108 | 6 | 108056589 | G | A | 1.00 | 0.02 | 0.37 | 1.65E-05 |
| rs72933109 | 6 | 108056646 | C | A | 1.00 | 0.02 | 0.37 | 1.65E-05 |
| rs72933110 | 6 | 108056725 | C | G | 1.00 | 0.02 | 0.37 | 1.66E-05 |
| rs78572573 | 6 | 108056792 | G | A | 1.00 | 0.02 | 0.37 | 1.66E-05 |
| rs56879234 | 7 | 152993373 | C | G | 0.99 | 0.02 | 0.40 | 1.66E-05 |
| rs35584137 | 3 | 141878096 | C | CA | 0.99 | 0.07 | 0.61 | 1.70E-05 |
| rs142565690 | 2 | 16145445 | CCTCCCT | C | 0.99 | 0.36 | 0.77 | 1.70E-05 |
| rs139706493 | 4 | 127961259 | CA | C | 0.99 | 0.03 | 21.69 | 1.74E-05 |
| rs56010936 | 4 | 127961261 | G | C | 0.99 | 0.03 | 21.69 | 1.74E-05 |
| rs56035350 | 3 | 55303163 | G | A | 0.97 | 0.20 | 13.66 | 1.76E-05 |
| rs34001849 | 3 | 141880005 | G | A | 0.99 | 0.07 | 0.60 | 1.76E-05 |
| rs2828148 | 21 | 24810388 | A | C | 0.97 | 0.35 | 0.77 | 1.78E-05 |
| rs77371062 | 4 | 7767098 | C | T | 1.00 | 0.04 | 0.52 | 1.81E-05 |
| rs77265165 | 5 | 139320824 | A | G | 0.96 | 0.09 | 15.30 | 1.82E-05 |
| chr15:98508249 | 15 | 98508249 | T | C | 1.00 | 0.01 | 36.52 | 1.84E-05 |
| rs868227 | 8 | 126263470 | A | G | 1.00 | 0.09 | 15.76 | 1.85E-05 |
| rs13264500 | 8 | 126213089 | T | C | 0.99 | 0.09 | 15.79 | 1.87E-05 |
| rs73994540 | 2 | 228684223 | G | A | 0.99 | 0.03 | 0.48 | 1.92E-05 |
| chr12:13446437 | 12 | 13446437 | G | GT | 0.98 | 0.18 | 0.72 | 1.92E-05 |
| rs143930926 | 15 | 98547251 | A | C | 1.00 | 0.01 | 40.48 | 1.94E-05 |
| rs72779201 | 2 | 8406702 | T | C | 0.99 | 0.07 | 16.13 | 1.95E-05 |
| rs144110335 | 15 | 98542653 | C | T | 1.00 | 0.01 | 41.13 | 1.95E-05 |
| rs7212267 | 17 | 13176356 | A | G | 1.00 | 0.26 | 0.74 | 2.02E-05 |
| rs72781009 | 2 | 8407845 | A | G | 0.99 | 0.07 | 16.11 | 2.04E-05 |
| rs56129208 | 2 | 8408180 | A | G | 0.99 | 0.07 | 22.28 | 2.07E-05 |
| rs901576 | 5 | 113449360 | A | T | 0.98 | 0.47 | 12.91 | 2.14E-05 |
| rs75646686 | 7 | 152994478 | G | A | 0.99 | 0.02 | 0.41 | 2.14E-05 |
| rs183996049 | 17 | 78370516 | A | G | 0.99 | 0.02 | 27.84 | 2.15E-05 |
| rs72779189 | 2 | 8395447 | A | G | 1.00 | 0.06 | 16.95 | 2.17E-05 |
| rs80291999 | 7 | 152994434 | C | T | 0.99 | 0.02 | 0.41 | 2.19E-05 |
| rs10274315 | 7 | 138705619 | A | G | 0.90 | 0.25 | 0.77 | 2.20E-05 |
| rs118072351 | 13 | 95912610 | C | A | 1.00 | 0.05 | 18.72 | 2.21E-05 |
| rs12943654 | 17 | 34451005 | T | C | 0.99 | 0.16 | 14.14 | 2.21E-05 |
| rs1105565 | 9 | 114722273 | A | C | 1.00 | 0.26 | 0.74 | 2.21E-05 |
| rs1105566 | 9 | 114722285 | C | A | 1.00 | 0.26 | 0.74 | 2.22E-05 |
| rs7981150 | 13 | 95288336 | G | C | 0.97 | 0.32 | 13.13 | 2.23E-05 |
| rs1105564 | 9 | 114722246 | G | T | 1.00 | 0.26 | 0.74 | 2.25E-05 |
| rs138028585 | 6 | 108056664 | G | GT | 1.00 | 0.02 | 0.38 | 2.26E-05 |
| rs7859340 | 9 | 114721272 | G | A | 1.00 | 0.26 | 0.74 | 2.27E-05 |
| rs140131954 | 15 | 98548359 | G | A | 1.00 | 0.01 | 40.08 | 2.28E-05 |
| rs55909940 | 2 | 16144820 | G | C | 0.99 | 0.36 | 0.77 | 2.29E-05 |
| rs2146822 | 13 | 95293574 | T | C | 1.00 | 0.27 | 13.39 | 2.30E-05 |
| rs77480497 | 15 | 98511210 | A | G | 1.00 | 0.01 | 38.26 | 2.33E-05 |
| rs1889224 | 9 | 114721683 | G | T | 1.00 | 0.26 | 0.74 | 2.33E-05 |
| rs146115587 | 5 | 42866619 | T | C | 0.96 | 0.09 | 0.67 | 2.35E-05 |
| rs141487254 | 15 | 98510808 | CT | C | 1.00 | 0.01 | 38.16 | 2.35E-05 |
| rs17103838 | 10 | 86436363 | A | G | 1.00 | 0.31 | 13.22 | 2.36E-05 |
| rs150073081 | 15 | 98517727 | G | A | 1.00 | 0.01 | 39.46 | 2.36E-05 |
| rs2418182 | 9 | 114722888 | T | C | 1.00 | 0.26 | 0.74 | 2.36E-05 |
| rs35955519 | 8 | 126256027 | G | T | 1.00 | 0.09 | 15.88 | 2.42E-05 |
| rs35336507 | 8 | 126256018 | A | G | 1.00 | 0.09 | 15.88 | 2.42E-05 |
| rs60348499 | 4 | 58888309 | C | A | 0.93 | 0.44 | 12.78 | 2.43E-05 |
| rs138767036 | 15 | 98515133 | G | T | 1.00 | 0.01 | 39.17 | 2.44E-05 |
| rs116816502 | 4 | 7767507 | G | A | 1.00 | 0.04 | 0.52 | 2.45E-05 |
| rs11026140 | 11 | 21649802 | G | A | 0.99 | 0.02 | 0.42 | 2.45E-05 |
| rs142043372 | 17 | 13175961 | G | GC | 1.00 | 0.26 | 0.75 | 2.46E-05 |
| chr17:13175962 | 17 | 13175962 | A | T | 1.00 | 0.26 | 0.75 | 2.46E-05 |
| rs137932788 | 2 | 16134768 | T | TTCTTTC | 0.90 | 0.48 | 0.79 | 2.48E-05 |
| rs79160406 | 6 | 7662089 | T | C | 0.98 | 0.03 | 19.08 | 2.50E-05 |
| rs12675943 | 8 | 126260092 | G | A | 1.00 | 0.09 | 15.88 | 2.52E-05 |
| rs4979066 | 9 | 114723688 | C | T | 1.00 | 0.26 | 0.74 | 2.52E-05 |
| rs11593101 | 10 | 86448366 | G | A | 1.00 | 0.31 | 13.20 | 2.53E-05 |
| rs9893201 | 17 | 34451277 | G | A | 0.99 | 0.16 | 14.06 | 2.54E-05 |
| rs11201173 | 10 | 86454472 | C | T | 1.00 | 0.31 | 13.19 | 2.55E-05 |
| rs11201170 | 10 | 86445410 | G | C | 1.00 | 0.31 | 11.69 | 2.55E-05 |
| rs59213736 | 10 | 86447497 | G | T | 1.00 | 0.31 | 13.20 | 2.55E-05 |
| rs111237282 | 1 | 217111259 | AT | A | 0.97 | 0.25 | 13.43 | 2.55E-05 |
| rs11201171 | 10 | 86446410 | A | C | 1.00 | 0.31 | 11.69 | 2.57E-05 |
| rs4573626 | 10 | 86439849 | C | T | 1.00 | 0.31 | 13.20 | 2.60E-05 |
| chr15:98514029 | 15 | 98514029 | TTTTGTCATCCGTTCATTA | T | 1.00 | 0.01 | 3.87 | 2.62E-05 |
| rs57139325 | 2 | 3519283 | T | C | 1.00 | 0.17 | 14.00 | 2.62E-05 |
| rs34737949 | 8 | 126127964 | G | A | 1.00 | 0.08 | 15.99 | 2.62E-05 |
| rs13267325 | 8 | 126129348 | C | T | 1.00 | 0.08 | 16.00 | 2.63E-05 |
| rs72779193 | 2 | 8403566 | G | A | 1.00 | 0.06 | 16.93 | 2.64E-05 |
| rs35861494 | 8 | 126133108 | G | A | 1.00 | 0.08 | 16.01 | 2.67E-05 |
| rs10739329 | 9 | 114720538 | A | G | 0.98 | 0.26 | 0.75 | 2.68E-05 |
| rs61741967 | 15 | 98513169 | C | T | 1.00 | 0.01 | 38.42 | 2.68E-05 |
| rs67890826 | 2 | 16146989 | C | T | 0.98 | 0.36 | 0.77 | 2.81E-05 |
| rs79333805 | 15 | 98510568 | G | A | 1.00 | 0.01 | 3.78 | 2.81E-05 |
| rs74918419 | 15 | 98510449 | T | C | 1.00 | 0.01 | 37.78 | 2.82E-05 |
| rs78704139 | 15 | 98510445 | C | A | 1.00 | 0.01 | 37.77 | 2.82E-05 |
| rs11625261 | 14 | 20975794 | T | A | 0.95 | 0.21 | 0.75 | 2.84E-05 |
| rs10179340 | 2 | 3523324 | A | G | 0.99 | 0.16 | 14.05 | 2.86E-05 |
| rs150473045 | 15 | 98556049 | T | C | 1.00 | 0.01 | 38.77 | 2.87E-05 |
| rs3981301 | 2 | 115204611 | A | ATT | 0.94 | 0.40 | 0.78 | 2.87E-05 |
| rs113922629 | 15 | 98510690 | CAT | C | 1.00 | 0.01 | 37.45 | 2.90E-05 |
| rs71465866 | 12 | 113339227 | T | C | 0.99 | 0.02 | 0.44 | 2.92E-05 |
| rs6768481 | 3 | 23696842 | C | T | 1.00 | 0.20 | 13.69 | 2.92E-05 |
| rs180793172 | 15 | 98556651 | A | G | 1.00 | 0.01 | 38.66 | 2.93E-05 |
| rs4601329 | 8 | 126254561 | T | C | 1.00 | 0.09 | 15.81 | 2.94E-05 |
| rs2418181 | 9 | 114723078 | A | C | 1.00 | 0.26 | 0.75 | 2.96E-05 |
| rs200222020 | 9 | 114723082 | AC | A | 1.00 | 0.26 | 0.75 | 2.96E-05 |
| rs12681602 | 8 | 126268020 | A | G | 1.00 | 0.10 | 15.38 | 2.97E-05 |
| rs142438580 | 16 | 78328305 | ACCT | A | 1.00 | 0.23 | 0.74 | 2.98E-05 |
| rs2063197 | 17 | 75300401 | G | A | 1.00 | 0.16 | 14.13 | 2.98E-05 |
| rs11869275 | 17 | 13173645 | G | A | 0.99 | 0.25 | 0.75 | 3.00E-05 |
| rs11405041 | 17 | 13175970 | T | TG | 1.00 | 0.26 | 0.75 | 3.01E-05 |
| rs13137436 | 4 | 177267049 | G | A | 1.00 | 0.06 | 0.59 | 3.02E-05 |
| rs13026141 | 2 | 2620052 | G | A | 0.99 | 0.14 | 1.42 | 3.03E-05 |
| rs10041275 | 5 | 153062161 | T | C | 1.00 | 0.25 | 0.75 | 3.03E-05 |
| rs2845768 | 21 | 37854364 | C | T | 1.00 | 0.24 | 13.37 | 3.04E-05 |
| rs139192410 | 15 | 98557863 | C | G | 1.00 | 0.01 | 38.48 | 3.05E-05 |
| rs116602014 | 4 | 177268988 | C | T | 1.00 | 0.06 | 0.59 | 3.06E-05 |
| rs35743587 | 4 | 177269199 | G | A | 1.00 | 0.06 | 0.59 | 3.06E-05 |
| rs112933470 | 4 | 177270357 | T | A | 1.00 | 0.06 | 0.59 | 3.08E-05 |
| rs219738 | 21 | 37856682 | T | C | 1.00 | 0.24 | 13.37 | 3.10E-05 |
| rs66826725 | 8 | 126169016 | A | G | 1.00 | 0.08 | 15.92 | 3.11E-05 |
| rs11201174 | 10 | 86454666 | A | G | 1.00 | 0.31 | 13.15 | 3.12E-05 |
| rs72720550 | 8 | 126170231 | A | G | 1.00 | 0.08 | 15.92 | 3.12E-05 |
| rs35564243 | 8 | 126171569 | G | A | 1.00 | 0.08 | 15.92 | 3.12E-05 |
| rs35774093 | 8 | 126171645 | T | C | 1.00 | 0.08 | 15.92 | 3.12E-05 |
| rs13277490 | 8 | 126153252 | C | T | 1.00 | 0.08 | 15.91 | 3.12E-05 |
| rs58241047 | 8 | 126151945 | AC | A | 1.00 | 0.08 | 15.91 | 3.12E-05 |
| rs34461511 | 8 | 126156120 | T | C | 1.00 | 0.08 | 15.91 | 3.12E-05 |
| rs13259062 | 8 | 126175858 | C | T | 1.00 | 0.08 | 15.92 | 3.12E-05 |
| rs13273231 | 8 | 126147516 | T | C | 1.00 | 0.08 | 15.91 | 3.12E-05 |
| rs189084708 | 15 | 98558718 | C | A | 1.00 | 0.01 | 38.33 | 3.14E-05 |
| rs12370755 | 12 | 16664330 | A | G | 1.00 | 0.48 | 12.83 | 3.14E-05 |
| rs35140299 | 8 | 126146957 | C | T | 1.00 | 0.08 | 15.90 | 3.19E-05 |
| rs2235394 | 6 | 11763351 | T | G | 1.00 | 0.41 | 12.88 | 3.19E-05 |
| rs148235576 | 8 | 76529921 | A | G | 1.00 | 0.02 | 0.36 | 3.19E-05 |
| rs199631128 | 14 | 28634610 | CTCA | C | 1.00 | 0.01 | 41.74 | 3.19E-05 |
| rs59878892 | 2 | 3519342 | G | A | 1.00 | 0.17 | 13.95 | 3.20E-05 |
| rs3092322 | 20 | 45416465 | A | T | 0.97 | 0.36 | 0.77 | 3.20E-05 |
| rs11026127 | 11 | 21554868 | C | T | 1.00 | 0.02 | 0.40 | 3.20E-05 |
| rs71563620 | 7 | 114870872 | G | A | 0.99 | 0.02 | 22.75 | 3.21E-05 |
| rs79791567 | 8 | 126187970 | AGAG | A | 1.00 | 0.08 | 15.89 | 3.23E-05 |
| rs4477044 | 8 | 126229570 | T | A | 1.00 | 0.08 | 15.88 | 3.24E-05 |
| rs17401283 | 8 | 126180331 | A | T | 1.00 | 0.08 | 1.59 | 3.25E-05 |
| rs34427316 | 8 | 126186427 | T | A | 1.00 | 0.08 | 15.89 | 3.26E-05 |
| rs35475899 | 8 | 126187075 | A | G | 1.00 | 0.08 | 15.89 | 3.26E-05 |
| rs2384882 | 8 | 126190444 | C | A | 1.00 | 0.08 | 15.89 | 3.26E-05 |
| rs35822368 | 8 | 126189156 | G | T | 1.00 | 0.08 | 15.89 | 3.26E-05 |
| rs13254947 | 8 | 126198584 | A | G | 1.00 | 0.08 | 15.89 | 3.26E-05 |
| rs13273421 | 8 | 126191572 | C | T | 1.00 | 0.08 | 15.89 | 3.26E-05 |
| rs34110249 | 8 | 126204459 | A | G | 1.00 | 0.08 | 15.89 | 3.26E-05 |
| rs34448805 | 8 | 126203574 | A | G | 1.00 | 0.08 | 15.89 | 3.26E-05 |
| rs146990265 | 8 | 126211868 | G | A | 1.00 | 0.08 | 15.89 | 3.26E-05 |
| rs4360308 | 8 | 126202988 | G | A | 1.00 | 0.08 | 15.89 | 3.26E-05 |
| rs56135036 | 8 | 126219876 | C | T | 1.00 | 0.08 | 15.89 | 3.26E-05 |
| rs149151217 | 8 | 126210858 | C | T | 1.00 | 0.08 | 15.89 | 3.26E-05 |
| rs34246020 | 8 | 126246328 | G | A | 1.00 | 0.08 | 15.88 | 3.27E-05 |
| rs112824993 | 8 | 126227755 | G | T | 1.00 | 0.08 | 15.89 | 3.27E-05 |
| rs66561774 | 8 | 126240333 | A | G | 1.00 | 0.08 | 15.89 | 3.27E-05 |
| rs35991395 | 8 | 126239472 | C | T | 1.00 | 0.08 | 15.89 | 3.27E-05 |
| rs16900452 | 8 | 126243100 | G | A | 1.00 | 0.08 | 15.89 | 3.27E-05 |
| rs34298715 | 8 | 126228373 | T | G | 1.00 | 0.08 | 15.89 | 3.27E-05 |
| rs35033076 | 8 | 126231204 | G | C | 1.00 | 0.08 | 15.89 | 3.27E-05 |
| rs71516769 | 8 | 126230876 | A | G | 1.00 | 0.08 | 15.89 | 3.27E-05 |
| rs17310558 | 8 | 126239155 | C | G | 1.00 | 0.08 | 15.89 | 3.27E-05 |
| rs77407761 | 8 | 126217445 | A | G | 1.00 | 0.08 | 15.89 | 3.27E-05 |
| rs35287869 | 8 | 126245089 | AG | A | 1.00 | 0.08 | 15.89 | 3.27E-05 |
| rs9894157 | 17 | 34451649 | G | A | 0.99 | 0.16 | 14.00 | 3.27E-05 |
| rs3092708 | 20 | 45416179 | A | T | 0.97 | 0.36 | 0.77 | 3.31E-05 |
| rs72781011 | 2 | 8412529 | T | C | 0.99 | 0.07 | 15.96 | 3.32E-05 |
| rs7174688 | 15 | 98505544 | G | C | 1.00 | 0.01 | 36.73 | 3.32E-05 |
| rs72781014 | 2 | 8413927 | T | C | 0.99 | 0.07 | 15.95 | 3.32E-05 |
| rs5746679 | 22 | 17080378 | A | G | 1.00 | 0.13 | 14.43 | 3.34E-05 |
| rs34634405 | 4 | 177267582 | C | T | 1.00 | 0.06 | 0.59 | 3.35E-05 |
| rs11150055 | 16 | 78326524 | T | A | 1.00 | 0.23 | 0.74 | 3.36E-05 |
| rs9892668 | 17 | 34451028 | G | A | 0.99 | 0.16 | 14.01 | 3.40E-05 |
| rs35401436 | 8 | 126185276 | T | A | 1.00 | 0.08 | 15.85 | 3.41E-05 |
| rs35215140 | 8 | 126200988 | C | T | 1.00 | 0.08 | 15.85 | 3.42E-05 |
| rs2049660 | 6 | 108055143 | G | A | 1.00 | 0.02 | 0.36 | 3.42E-05 |
| rs55937624 | 2 | 8388038 | G | A | 1.00 | 0.06 | 16.71 | 3.42E-05 |
| rs113228157 | 15 | 98504530 | C | T | 1.00 | 0.01 | 36.56 | 3.43E-05 |
| rs192769497 | 15 | 98503918 | T | G | 1.00 | 0.01 | 36.44 | 3.46E-05 |
| rs219749 | 21 | 37848438 | T | C | 1.00 | 0.31 | 13.07 | 3.47E-05 |
| rs219748 | 21 | 37848479 | C | T | 1.00 | 0.31 | 13.07 | 3.50E-05 |
| rs10046826 | 9 | 114725910 | A | T | 0.99 | 0.24 | 0.74 | 3.51E-05 |
| rs148751793 | 8 | 76481121 | T | C | 1.00 | 0.02 | 0.36 | 3.53E-05 |
| rs11383874 | 3 | 141885488 | G | GC | 1.00 | 0.07 | 0.61 | 3.56E-05 |
| rs219739 | 21 | 37856536 | G | A | 1.00 | 0.24 | 13.35 | 3.56E-05 |
| rs72781016 | 2 | 8416220 | A | G | 0.99 | 0.07 | 15.92 | 3.59E-05 |
| rs16866656 | 2 | 8416174 | T | C | 0.99 | 0.07 | 15.92 | 3.59E-05 |
| rs62207502 | 20 | 31596646 | G | A | 0.99 | 0.04 | 0.52 | 3.59E-05 |
| rs16866655 | 2 | 8416094 | A | C | 0.99 | 0.07 | 15.92 | 3.61E-05 |
| rs17447180 | 7 | 83171681 | C | T | 1.00 | 0.12 | 14.66 | 3.61E-05 |
| rs927014 | 6 | 106893293 | T | C | 1.00 | 0.41 | 1.29 | 3.61E-05 |
| rs927015 | 6 | 106893170 | A | T | 1.00 | 0.41 | 1.29 | 3.61E-05 |
| rs6908605 | 6 | 106893041 | T | C | 1.00 | 0.41 | 1.29 | 3.61E-05 |
| rs6928410 | 6 | 106892991 | A | G | 1.00 | 0.41 | 1.29 | 3.62E-05 |
| rs140883485 | 6 | 106892856 | A | AAAAC | 1.00 | 0.41 | 12.86 | 3.62E-05 |
| rs2850112 | 21 | 37854507 | A | G | 1.00 | 0.24 | 13.33 | 3.65E-05 |
| rs4433392 | 1 | 116209709 | T | C | 1.00 | 0.37 | 0.78 | 3.65E-05 |
| rs11385581 | 6 | 231085 | A | AT | 0.95 | 0.33 | 0.78 | 3.68E-05 |
| rs9503021 | 6 | 231638 | G | T | 0.97 | 0.32 | 0.77 | 3.76E-05 |
| rs2850111 | 21 | 37853812 | G | A | 1.00 | 0.24 | 13.32 | 3.81E-05 |
| rs199704995 | 2 | 8402794 | C | CA | 1.00 | 0.06 | 16.87 | 3.85E-05 |
| rs6749249 | 2 | 3507275 | C | T | 0.99 | 0.17 | 0.72 | 3.85E-05 |
| rs145559632 | 4 | 57323736 | G | A | 0.99 | 0.02 | 0.41 | 3.90E-05 |
| rs34313605 | 8 | 126271948 | A | G | 1.00 | 0.09 | 15.38 | 3.92E-05 |
| rs33997248 | 9 | 125985328 | TGATA | T | 0.91 | 0.22 | 13.00 | 3.93E-05 |
| rs112882257 | 3 | 141882107 | A | G | 1.00 | 0.07 | 0.61 | 3.94E-05 |
| rs7998457 | 13 | 95288188 | G | A | 0.97 | 0.31 | 13.03 | 4.00E-05 |
| rs10490219 | 2 | 8400895 | C | T | 1.00 | 0.06 | 16.83 | 4.00E-05 |
| rs2633324 | 21 | 37854906 | G | A | 1.00 | 0.24 | 13.32 | 4.00E-05 |
| rs35856259 | 8 | 126244605 | C | CA | 0.99 | 0.09 | 15.30 | 4.01E-05 |
| rs8073447 | 17 | 34452832 | G | T | 0.99 | 0.16 | 13.95 | 4.04E-05 |
| rs7126227 | 11 | 36898896 | T | C | 0.98 | 0.28 | 0.76 | 4.05E-05 |
| rs66873334 | 2 | 15204426 | CAAAAAA | C | 0.99 | 0.31 | 0.76 | 4.07E-05 |
| rs112850403 | 19 | 515464 | G | A | 0.93 | 0.20 | 13.27 | 4.11E-05 |
| rs55983440 | 4 | 127621858 | T | C | 0.99 | 0.03 | 21.38 | 4.11E-05 |
| rs71070728 | 1 | 118915377 | C | CT | 0.93 | 0.24 | 13.12 | 4.14E-05 |
| rs34958083 | 6 | 11765369 | GT | G | 1.00 | 0.41 | 12.82 | 4.17E-05 |
| rs7766727 | 6 | 108055530 | A | G | 1.00 | 0.02 | 24.69 | 4.19E-05 |
| rs150963317 | 15 | 98567986 | T | C | 1.00 | 0.01 | 36.96 | 4.19E-05 |
| rs9897276 | 17 | 34453441 | C | T | 0.99 | 0.16 | 13.94 | 4.21E-05 |
| rs11872570 | 18 | 74090077 | G | A | 0.97 | 0.06 | 16.67 | 4.24E-05 |
| rs72720569 | 8 | 126258407 | G | T | 1.00 | 0.08 | 15.68 | 4.24E-05 |
| rs67929574 | 2 | 16132601 | C | T | 0.99 | 0.38 | 0.78 | 4.25E-05 |
| rs3213827 | 2 | 16137237 | G | A | 1.00 | 0.46 | 0.78 | 4.26E-05 |
| rs140602480 | 15 | 98551893 | T | G | 1.00 | 0.01 | 36.10 | 4.27E-05 |
| rs7588144 | 2 | 16132558 | C | T | 0.99 | 0.38 | 0.78 | 4.27E-05 |
| rs73138311 | 3 | 23697010 | A | G | 0.97 | 0.18 | 13.62 | 4.32E-05 |
| rs8070275 | 17 | 22142172 | T | C | 1.00 | 0.34 | 12.96 | 4.33E-05 |
| rs115936014 | 3 | 141882139 | C | T | 1.00 | 0.07 | 0.61 | 4.36E-05 |
| rs78214974 | 13 | 106941417 | G | A | 0.98 | 0.04 | 18.22 | 4.41E-05 |
| rs10026330 | 4 | 57103784 | A | T | 0.94 | 0.38 | 0.79 | 4.43E-05 |
| rs145057453 | 10 | 13256125 | G | A | 0.99 | 0.01 | 0.36 | 4.44E-05 |
| rs10424744 | 19 | 51520924 | T | G | 0.99 | 0.29 | 13.07 | 4.45E-05 |
| rs8074386 | 17 | 34452725 | C | T | 0.99 | 0.16 | 13.92 | 4.46E-05 |
| rs12620727 | 2 | 3498883 | G | T | 1.00 | 0.16 | 0.71 | 4.48E-05 |
| rs11033890 | 11 | 36989149 | G | A | 1.00 | 0.33 | 12.98 | 4.52E-05 |
| rs13101604 | 4 | 177290105 | G | C | 0.99 | 0.05 | 0.59 | 4.53E-05 |
| rs115041907 | 4 | 7778155 | C | T | 1.00 | 0.05 | 0.55 | 4.53E-05 |
| rs145660239 | 18 | 6739854 | CT | C | 0.89 | 0.30 | 12.75 | 4.58E-05 |
| rs3955403 | 8 | 126229601 | A | ATT | 1.00 | 0.08 | 15.75 | 4.58E-05 |
| rs9634468 | 13 | 109439232 | C | T | 1.00 | 0.11 | 0.68 | 4.58E-05 |
| rs9287666 | 2 | 16136922 | T | C | 1.00 | 0.46 | 0.78 | 4.61E-05 |
| rs140930777 | 2 | 8395953 | CTTGT | C | 1.00 | 0.06 | 16.69 | 4.62E-05 |
| rs6841702 | 4 | 177280998 | C | T | 1.00 | 0.06 | 0.60 | 4.63E-05 |
| rs7299188 | 12 | 129774394 | C | G | 0.94 | 0.35 | 12.79 | 4.64E-05 |
| rs68066632 | 3 | 141884299 | GT | G | 1.00 | 0.07 | 0.61 | 4.65E-05 |
| rs201313838 | 2 | 233958813 | C | A | 0.99 | 0.02 | 0.39 | 4.65E-05 |
| rs9765131 | 5 | 41136248 | T | C | 0.96 | 0.28 | 13.01 | 4.71E-05 |
| rs9897261 | 17 | 34453424 | C | A | 0.99 | 0.16 | 13.90 | 4.71E-05 |
| rs219740 | 21 | 37853162 | C | T | 1.00 | 0.24 | 13.27 | 4.71E-05 |
| rs10423037 | 19 | 51520933 | A | C | 0.99 | 0.29 | 13.06 | 4.72E-05 |
| rs2845765 | 21 | 37853079 | G | C | 1.00 | 0.24 | 13.27 | 4.75E-05 |
| rs219741 | 21 | 37853036 | C | T | 1.00 | 0.24 | 13.27 | 4.77E-05 |
| rs141009116 | 22 | 38313557 | C | T | 0.99 | 0.03 | 20.32 | 4.78E-05 |
| rs150936982 | 12 | 121042154 | GAGAGCAT | G | 0.99 | 0.02 | 0.46 | 4.79E-05 |
| rs55760288 | 6 | 111388881 | C | T | 0.97 | 0.33 | 12.87 | 4.81E-05 |
| rs854719 | 7 | 94594055 | A | G | 0.98 | 0.08 | 0.65 | 4.82E-05 |
| rs79490092 | 14 | 33063691 | A | C | 0.99 | 0.03 | 0.46 | 4.83E-05 |
| rs1616164 | 8 | 13176985 | G | C | 0.90 | 0.36 | 1.26 | 4.90E-05 |
| rs13127237 | 4 | 177283245 | C | T | 1.00 | 0.06 | 0.60 | 4.92E-05 |
| rs76553368 | 17 | 75302388 | A | G | 0.99 | 0.09 | 15.20 | 4.97E-05 |
| rs35812428 | 8 | 126193049 | TA | T | 1.00 | 0.08 | 15.80 | 5.03E-05 |
| rs2420562 | 3 | 168366814 | T | A | 1.00 | 0.47 | 0.79 | 5.03E-05 |
| rs2868198 | 20 | 43367935 | C | T | 1.00 | 0.43 | 0.78 | 5.03E-05 |
| rs111562278 | 7 | 94695042 | C | T | 0.97 | 0.10 | 0.68 | 5.09E-05 |
| rs56125162 | 20 | 43367333 | GT | G | 1.00 | 0.46 | 0.78 | 5.14E-05 |
| rs73343343 | 20 | 24513383 | C | T | 1.00 | 0.01 | 0.30 | 5.20E-05 |
| rs2090123 | 10 | 61525567 | A | G | 0.97 | 0.46 | 0.79 | 5.22E-05 |
| rs6588565 | 1 | 56037324 | C | T | 0.98 | 0.08 | 15.65 | 5.25E-05 |
| rs34542632 | 17 | 13168561 | G | A | 0.99 | 0.24 | 0.75 | 5.25E-05 |
| rs219742 | 21 | 37852001 | T | C | 1.00 | 0.24 | 13.25 | 5.31E-05 |
| rs12585975 | 13 | 109443806 | T | G | 1.00 | 0.11 | 0.68 | 5.31E-05 |
| rs10423292 | 19 | 51521078 | A | C | 0.99 | 0.28 | 13.05 | 5.32E-05 |
| rs133932 | 22 | 33394092 | C | T | 1.00 | 0.02 | 24.03 | 5.34E-05 |
| rs17759015 | 2 | 114923129 | A | T | 0.99 | 0.04 | 18.91 | 5.34E-05 |
| rs11108731 | 12 | 97298802 | A | C | 1.00 | 0.01 | 0.35 | 5.34E-05 |
| rs10754329 | 1 | 116214111 | G | C | 1.00 | 0.33 | 0.77 | 5.37E-05 |
| rs9861268 | 3 | 191687812 | C | T | 1.00 | 0.46 | 12.82 | 5.38E-05 |
| rs55980070 | 2 | 3514866 | C | T | 1.00 | 0.16 | 0.72 | 5.38E-05 |
| rs4978472 | 9 | 114725018 | T | C | 1.00 | 0.25 | 0.75 | 5.39E-05 |
| rs6928018 | 6 | 106892796 | A | G | 1.00 | 0.41 | 12.78 | 5.40E-05 |
| rs9559412 | 13 | 109444449 | G | A | 1.00 | 0.11 | 0.68 | 5.43E-05 |
| rs55791974 | 6 | 111387028 | A | G | 1.00 | 0.33 | 12.90 | 5.43E-05 |
| rs56024811 | 11 | 78715165 | C | A | 0.97 | 0.37 | 12.81 | 5.44E-05 |
| rs2235393 | 6 | 11763403 | G | C | 1.00 | 0.46 | 12.77 | 5.45E-05 |
| rs10793351 | 11 | 78714748 | T | C | 0.97 | 0.37 | 1.28 | 5.46E-05 |
| rs117987631 | 14 | 103313904 | T | G | 0.99 | 0.05 | 0.58 | 5.47E-05 |
| rs7277324 | 21 | 37858267 | C | G | 0.99 | 0.24 | 13.20 | 5.47E-05 |
| rs10929406 | 2 | 16136439 | T | C | 1.00 | 0.46 | 0.78 | 5.51E-05 |
| rs7996889 | 13 | 103220683 | C | T | 1.00 | 0.36 | 12.85 | 5.53E-05 |
| rs1818692 | 17 | 22121729 | G | T | 1.00 | 0.34 | 12.90 | 5.54E-05 |
| rs943671 | 13 | 103219454 | T | C | 1.00 | 0.36 | 12.85 | 5.56E-05 |
| rs17591272 | 13 | 103219888 | G | T | 1.00 | 0.36 | 12.85 | 5.57E-05 |
| rs9585939 | 13 | 103203024 | C | T | 1.00 | 0.37 | 12.83 | 5.58E-05 |
| rs943672 | 13 | 103219592 | C | T | 1.00 | 0.36 | 12.85 | 5.59E-05 |
| rs62248549 | 3 | 55272672 | C | T | 0.97 | 0.21 | 13.37 | 5.59E-05 |
| rs943670 | 13 | 103219408 | G | A | 1.00 | 0.36 | 12.85 | 5.60E-05 |
| rs943669 | 13 | 103219191 | C | A | 1.00 | 0.36 | 12.85 | 5.61E-05 |
| rs12871233 | 13 | 109425455 | G | A | 1.00 | 0.08 | 0.64 | 5.63E-05 |
| rs35323001 | 13 | 103219898 | C | T | 1.00 | 0.36 | 12.85 | 5.67E-05 |
| rs11793712 | 9 | 130181069 | C | T | 1.00 | 0.13 | 14.44 | 5.68E-05 |
| rs55896129 | 6 | 111386925 | G | T | 1.00 | 0.33 | 12.89 | 5.71E-05 |
| rs9303700 | 17 | 34450463 | G | T | 1.00 | 0.16 | 13.83 | 5.73E-05 |
| rs72651354 | 13 | 103293247 | G | A | 1.00 | 0.06 | 16.95 | 5.78E-05 |
| rs13319616 | 3 | 168380386 | G | T | 1.00 | 0.47 | 0.79 | 5.78E-05 |
| rs1427681 | 2 | 16150975 | G | A | 0.95 | 0.36 | 0.79 | 5.83E-05 |
| rs1927017 | 13 | 103219033 | T | G | 1.00 | 0.36 | 12.84 | 5.86E-05 |
| rs11620538 | 13 | 103210114 | T | G | 1.00 | 0.36 | 1.28 | 5.88E-05 |
| rs7814724 | 8 | 87691218 | A | G | 1.00 | 0.13 | 0.69 | 5.88E-05 |
| chr6:16731696 | 6 | 16731696 | T | C | 0.98 | 0.06 | 0.61 | 5.89E-05 |
| rs77364198 | 13 | 95906308 | C | T | 1.00 | 0.06 | 16.96 | 5.92E-05 |
| rs970633 | 8 | 87690748 | G | C | 1.00 | 0.13 | 0.69 | 5.94E-05 |
| rs58508627 | 6 | 82942872 | CAA | C | 0.98 | 0.23 | 0.76 | 5.95E-05 |
| rs75166455 | 13 | 95909678 | T | C | 1.00 | 0.06 | 16.96 | 5.95E-05 |
| rs113221335 | 6 | 108084807 | T | A | 1.00 | 0.02 | 0.39 | 5.98E-05 |
| rs10843990 | 12 | 31799859 | T | A | 0.95 | 0.47 | 0.79 | 6.00E-05 |
| rs1372171 | 8 | 87690145 | G | A | 1.00 | 0.13 | 0.69 | 6.02E-05 |
| rs11832997 | 12 | 16694800 | C | T | 1.00 | 0.41 | 0.78 | 6.06E-05 |
| rs72779171 | 2 | 8359538 | G | A | 0.99 | 0.06 | 16.32 | 6.07E-05 |
| rs11622053 | 14 | 101648535 | G | A | 0.95 | 0.16 | 13.56 | 6.07E-05 |
| rs140348357 | 5 | 135258030 | A | T | 0.99 | 0.02 | 22.49 | 6.07E-05 |
| rs149718931 | 5 | 135257977 | T | A | 0.99 | 0.02 | 22.49 | 6.08E-05 |
| rs219743 | 21 | 37851095 | A | C | 1.00 | 0.24 | 13.22 | 6.08E-05 |
| rs1372172 | 8 | 87688924 | G | A | 1.00 | 0.13 | 0.69 | 6.10E-05 |
| rs2113328 | 5 | 1379826 | G | A | 0.98 | 0.15 | 1.39 | 6.12E-05 |
| rs13139047 | 4 | 177244885 | T | C | 1.00 | 0.09 | 0.66 | 6.19E-05 |
| rs34485448 | 20 | 43370519 | T | G | 1.00 | 0.43 | 0.78 | 6.19E-05 |
| rs11129317 | 3 | 28312835 | C | T | 0.99 | 0.17 | 13.77 | 6.24E-05 |
| rs11502999 | 12 | 16678301 | C | T | 1.00 | 0.33 | 12.91 | 6.25E-05 |
| rs13392783 | 2 | 115198886 | A | G | 1.00 | 0.34 | 0.78 | 6.25E-05 |
| rs140771568 | 10 | 13264107 | G | C | 0.99 | 0.01 | 0.37 | 6.26E-05 |
| rs139147650 | 12 | 114621014 | C | T | 0.99 | 0.01 | 0.37 | 6.28E-05 |
| rs6841958 | 4 | 738512 | T | C | 0.96 | 0.21 | 13.25 | 6.28E-05 |
| rs75578539 | 12 | 13450244 | T | G | 1.00 | 0.16 | 0.72 | 6.29E-05 |
| rs148318007 | 1 | 175001710 | T | C | 0.99 | 0.04 | 0.54 | 6.30E-05 |
| rs183770089 | 10 | 59232099 | A | G | 0.99 | 0.01 | 0.38 | 6.30E-05 |
| rs7687558 | 4 | 55664102 | T | C | 1.00 | 0.18 | 13.59 | 6.30E-05 |
| rs137938530 | 10 | 59220906 | C | T | 0.99 | 0.01 | 0.38 | 6.31E-05 |
| rs55847508 | 5 | 42336102 | A | T | 0.99 | 0.05 | 0.60 | 6.35E-05 |
| rs16900462 | 8 | 126250796 | G | A | 0.96 | 0.12 | 14.24 | 6.39E-05 |
| rs1372173 | 8 | 87684674 | A | G | 1.00 | 0.13 | 0.69 | 6.39E-05 |
| rs6471482 | 8 | 87679303 | A | C | 1.00 | 0.12 | 0.69 | 6.40E-05 |
| chr1:6815348 | 1 | 6815348 | CA | C | 0.96 | 0.13 | 14.03 | 6.43E-05 |
| rs7754456 | 6 | 111387327 | A | G | 1.00 | 0.33 | 12.88 | 6.44E-05 |
| rs56369430 | 2 | 3514275 | C | T | 1.00 | 0.16 | 0.72 | 6.45E-05 |
| rs11108728 | 12 | 97288694 | A | G | 1.00 | 0.01 | 0.35 | 6.51E-05 |
| rs11873047 | 18 | 11263133 | G | A | 0.95 | 0.18 | 0.75 | 6.52E-05 |
| rs114252047 | 5 | 42347260 | T | C | 0.99 | 0.05 | 0.60 | 6.54E-05 |
| chr10:13254386 | 10 | 13254386 | G | A | 0.99 | 0.01 | 0.37 | 6.55E-05 |
| rs17389911 | 2 | 8365872 | A | G | 0.99 | 0.06 | 16.24 | 6.59E-05 |
| rs188733 | 21 | 37851975 | C | G | 1.00 | 0.24 | 13.19 | 6.61E-05 |
| rs72779172 | 2 | 8361400 | G | C | 0.99 | 0.06 | 16.34 | 6.63E-05 |
| rs12992556 | 2 | 3506519 | C | T | 1.00 | 0.16 | 0.72 | 6.66E-05 |
| rs7583568 | 2 | 3515708 | C | T | 1.00 | 0.16 | 13.85 | 6.67E-05 |
| rs2169956 | 17 | 25470744 | T | A | 1.00 | 0.36 | 12.84 | 6.68E-05 |
| rs56017562 | 12 | 13445843 | C | A | 1.00 | 0.16 | 0.72 | 6.70E-05 |
| rs6471487 | 8 | 87682318 | T | C | 1.00 | 0.13 | 0.70 | 6.71E-05 |
| rs10190397 | 2 | 3516085 | C | T | 1.00 | 0.16 | 13.84 | 6.71E-05 |
| rs2127282 | 17 | 25470790 | G | A | 1.00 | 0.36 | 1.28 | 6.73E-05 |
| rs2046021 | 17 | 25470461 | G | C | 1.00 | 0.36 | 1.28 | 6.73E-05 |
| rs6505102 | 17 | 25471264 | A | G | 1.00 | 0.36 | 1.28 | 6.73E-05 |
| rs11650311 | 17 | 25473017 | A | G | 1.00 | 0.36 | 1.28 | 6.73E-05 |
| rs71048001 | 11 | 36898895 | G | GCGTCTC | 0.98 | 0.27 | 13.03 | 6.80E-05 |
| rs34099505 | 8 | 79618856 | T | C | 0.99 | 0.13 | 0.70 | 6.82E-05 |
| rs219744 | 21 | 37850270 | G | A | 0.99 | 0.29 | 12.98 | 6.83E-05 |
| rs219745 | 21 | 37850143 | C | T | 0.99 | 0.29 | 12.98 | 6.84E-05 |
| rs219747 | 21 | 37849885 | G | A | 0.99 | 0.29 | 12.98 | 6.84E-05 |
| rs219746 | 21 | 37850027 | G | A | 0.99 | 0.29 | 12.98 | 6.84E-05 |
| rs11365554 | 21 | 37849943 | TC | T | 0.99 | 0.29 | 12.98 | 6.84E-05 |
| rs36014448 | 1 | 232052135 | G | A | 0.99 | 0.02 | 22.02 | 6.93E-05 |
| chr5:139389880 | 5 | 139389880 | C | CA | 0.95 | 0.15 | 13.74 | 6.94E-05 |
| rs62434794 | 6 | 156463532 | T | C | 0.98 | 0.15 | 13.96 | 6.94E-05 |
| rs12052249 | 2 | 3511044 | C | A | 1.00 | 0.16 | 0.72 | 6.96E-05 |
| rs147079632 | 3 | 168359193 | T | TA | 0.99 | 0.32 | 0.78 | 6.97E-05 |
| rs765504 | 5 | 133827455 | G | C | 0.90 | 0.38 | 12.53 | 6.98E-05 |
| rs11470946 | 20 | 59529288 | TCTC | T | 0.98 | 0.09 | 15.12 | 7.00E-05 |
| rs71440003 | 13 | 109439253 | T | A | 1.00 | 0.08 | 0.64 | 7.03E-05 |
| rs73074879 | 7 | 23591209 | G | A | 0.98 | 0.07 | 0.64 | 7.04E-05 |
| rs66929016 | 8 | 126230096 | C | T | 1.00 | 0.08 | 15.59 | 7.04E-05 |
| rs34320491 | 14 | 73274127 | T | A | 0.99 | 0.24 | 0.75 | 7.05E-05 |
| rs62207503 | 20 | 31597323 | C | T | 0.99 | 0.04 | 0.53 | 7.05E-05 |
| rs12328995 | 2 | 3504073 | G | A | 1.00 | 0.16 | 0.72 | 7.06E-05 |
| rs35239575 | 2 | 3508567 | G | T | 1.00 | 0.16 | 0.72 | 7.20E-05 |
| rs7161536 | 14 | 23255210 | C | A | 0.97 | 0.06 | 0.63 | 7.24E-05 |
| rs80347742 | 6 | 21375136 | A | G | 1.00 | 0.02 | 27.63 | 7.24E-05 |
| rs149154033 | 13 | 103336888 | G | C | 1.00 | 0.06 | 16.92 | 7.28E-05 |
| rs58350940 | 2 | 151722436 | C | T | 0.92 | 0.22 | 0.77 | 7.28E-05 |
| rs12452982 | 17 | 22216558 | A | C | 1.00 | 0.34 | 12.86 | 7.28E-05 |
| rs1842168 | 17 | 25543064 | C | T | 0.97 | 0.35 | 12.78 | 7.30E-05 |
| rs10508094 | 13 | 103333413 | C | G | 1.00 | 0.06 | 16.92 | 7.33E-05 |
| rs1814029 | 17 | 22214259 | C | T | 1.00 | 0.34 | 12.86 | 7.33E-05 |
| rs10772945 | 12 | 16692603 | T | G | 1.00 | 0.41 | 12.76 | 7.35E-05 |
| rs11686974 | 2 | 3527368 | A | G | 0.99 | 0.16 | 13.78 | 7.37E-05 |
| rs1913800 | 17 | 22181268 | C | T | 1.00 | 0.34 | 12.85 | 7.37E-05 |
| rs4889745 | 17 | 22124025 | C | T | 1.00 | 0.34 | 12.85 | 7.38E-05 |
| rs11127433 | 2 | 3503767 | T | C | 1.00 | 0.16 | 0.73 | 7.39E-05 |
| rs34781041 | 2 | 3513855 | G | A | 1.00 | 0.16 | 0.72 | 7.40E-05 |
| rs11384182 | 2 | 153024245 | G | GA | 0.97 | 0.34 | 0.78 | 7.40E-05 |
| rs36113402 | 2 | 3512754 | G | A | 1.00 | 0.16 | 0.72 | 7.40E-05 |
| rs76543037 | 14 | 47867975 | C | T | 1.00 | 0.03 | 20.22 | 7.41E-05 |
| rs12948980 | 17 | 22136891 | A | G | 1.00 | 0.34 | 12.85 | 7.42E-05 |
| rs11205386 | 1 | 150481738 | C | T | 1.00 | 0.11 | 14.76 | 7.44E-05 |
| rs4889749 | 17 | 22184866 | C | T | 1.00 | 0.34 | 12.85 | 7.44E-05 |
| rs35504778 | 3 | 28312573 | C | T | 0.99 | 0.17 | 13.75 | 7.46E-05 |
| rs1515750 | 17 | 22196513 | A | T | 1.00 | 0.34 | 1.29 | 7.47E-05 |
| chr13:103210034 | 13 | 103210034 | AT | A | 1.00 | 0.06 | 17.06 | 7.48E-05 |
| rs75846530 | 7 | 4554915 | C | T | 1.00 | 0.01 | 3.36 | 7.50E-05 |
| chr8:26911874 | 8 | 26911874 | C | CA | 0.98 | 0.10 | 0.68 | 7.50E-05 |
| rs10766344 | 11 | 16783545 | A | G | 0.95 | 0.40 | 0.79 | 7.53E-05 |
| rs67509453 | 17 | 22214260 | A | G | 1.00 | 0.34 | 12.85 | 7.56E-05 |
| rs8074936 | 17 | 22179322 | G | A | 1.00 | 0.34 | 12.85 | 7.57E-05 |
| rs62052534 | 17 | 25267077 | A | G | 0.88 | 0.45 | 12.42 | 7.59E-05 |
| rs1560996 | 11 | 78713662 | C | G | 0.98 | 0.37 | 12.75 | 7.60E-05 |
| rs140704676 | 8 | 126210744 | G | A | 0.99 | 0.09 | 15.33 | 7.68E-05 |
| rs142157943 | 17 | 22148411 | T | TG | 1.00 | 0.34 | 12.84 | 7.69E-05 |
| rs72779188 | 2 | 8393977 | C | T | 0.99 | 0.06 | 16.54 | 7.70E-05 |
| chr12:49772044 | 12 | 49772044 | G | GTTTAT | 0.99 | 0.05 | 1.69 | 7.71E-05 |
| rs8080557 | 17 | 22172228 | G | A | 1.00 | 0.34 | 12.85 | 7.71E-05 |
| rs8076631 | 17 | 22172118 | C | T | 1.00 | 0.34 | 12.85 | 7.71E-05 |
| rs35827677 | 4 | 177245488 | T | G | 1.00 | 0.09 | 0.66 | 7.72E-05 |
| rs6025318 | 20 | 55606804 | C | T | 1.00 | 0.33 | 12.84 | 7.73E-05 |
| rs35882099 | 13 | 109441503 | T | C | 1.00 | 0.08 | 0.64 | 7.74E-05 |
| rs12165036 | 17 | 22169878 | C | G | 1.00 | 0.34 | 12.84 | 7.78E-05 |
| rs71594066 | 4 | 174696434 | A | AT | 0.96 | 0.17 | 13.49 | 7.79E-05 |
| rs61844353 | 10 | 12134790 | G | A | 1.00 | 0.29 | 0.77 | 7.81E-05 |
| rs71371102 | 17 | 22166358 | A | T | 1.00 | 0.34 | 12.84 | 7.82E-05 |
| rs143387607 | 4 | 57374846 | C | T | 0.99 | 0.02 | 0.43 | 7.83E-05 |
| rs7671099 | 4 | 177286364 | C | T | 1.00 | 0.06 | 0.61 | 7.84E-05 |
| rs6073534 | 20 | 43365504 | C | T | 1.00 | 0.42 | 0.79 | 7.87E-05 |
| rs9634572 | 13 | 109426194 | C | A | 1.00 | 0.12 | 0.69 | 7.88E-05 |
| rs28531087 | 17 | 22116338 | A | G | 1.00 | 0.34 | 12.83 | 7.90E-05 |
| rs9570134 | 13 | 60036527 | C | T | 0.99 | 0.19 | 1.35 | 7.91E-05 |
| rs4578351 | 11 | 16587580 | T | C | 0.97 | 0.28 | 0.77 | 7.95E-05 |
| rs2249234 | 21 | 37850468 | A | G | 1.00 | 0.24 | 13.16 | 7.96E-05 |
| rs7993655 | 13 | 103204605 | C | T | 1.00 | 0.37 | 1.28 | 7.96E-05 |
| rs6841674 | 4 | 78334882 | C | T | 1.00 | 0.33 | 12.83 | 8.01E-05 |
| rs76834393 | 5 | 133272516 | C | T | 0.99 | 0.01 | 28.88 | 8.06E-05 |
| rs57221077 | 2 | 16152645 | C | CA | 0.94 | 0.36 | 0.79 | 8.07E-05 |
| rs6707268 | 2 | 3506980 | A | G | 1.00 | 0.16 | 0.73 | 8.08E-05 |
| rs7141689 | 14 | 69371155 | T | A | 0.99 | 0.17 | 0.73 | 8.08E-05 |
| rs34534512 | 2 | 3508800 | T | C | 1.00 | 0.16 | 0.73 | 8.09E-05 |
| rs6908055 | 6 | 106909194 | A | G | 1.00 | 0.18 | 13.73 | 8.09E-05 |
| rs75508402 | 9 | 125182723 | G | T | 0.93 | 0.16 | 0.74 | 8.11E-05 |
| rs7210668 | 17 | 22168795 | A | G | 1.00 | 0.34 | 12.82 | 8.13E-05 |
| rs67120114 | 2 | 3507075 | CACTG | C | 1.00 | 0.16 | 0.73 | 8.13E-05 |
| rs35029535 | 5 | 1284976 | C | T | 0.90 | 0.36 | 12.54 | 8.14E-05 |
| rs135084 | 22 | 43820681 | GT | G | 1.00 | 0.47 | 0.79 | 8.14E-05 |
| rs9582598 | 13 | 103204306 | A | G | 1.00 | 0.37 | 12.76 | 8.16E-05 |
| rs11658968 | 17 | 22144986 | G | T | 0.99 | 0.34 | 12.82 | 8.18E-05 |
| rs17089688 | 4 | 60403764 | C | G | 1.00 | 0.13 | 0.71 | 8.18E-05 |
| rs6422707 | 2 | 3519572 | A | C | 1.00 | 0.16 | 13.78 | 8.24E-05 |
| rs113833672 | 17 | 80076973 | C | T | 0.98 | 0.19 | 0.75 | 8.25E-05 |
| rs28797818 | 4 | 177286586 | C | T | 1.00 | 0.06 | 0.61 | 8.32E-05 |
| rs753249 | 4 | 57093548 | C | A | 1.00 | 0.39 | 0.79 | 8.32E-05 |
| rs4955617 | 3 | 168383190 | T | C | 1.00 | 0.32 | 0.78 | 8.32E-05 |
| rs11328817 | 19 | 6299823 | AT | TT | 0.98 | 0.05 | 0.59 | 8.33E-05 |
| rs34614434 | 17 | 22131179 | A | T | 1.00 | 0.34 | 12.82 | 8.33E-05 |
| rs7324355 | 13 | 95925235 | C | A | 1.00 | 0.06 | 16.72 | 8.33E-05 |
| rs6605294 | 2 | 3520047 | G | A | 1.00 | 0.16 | 13.78 | 8.35E-05 |
| rs34060970 | 17 | 22194839 | A | G | 0.99 | 0.34 | 12.82 | 8.36E-05 |
| rs1559780 | 4 | 187948625 | A | C | 1.00 | 0.38 | 0.78 | 8.36E-05 |
| rs2954358 | 5 | 113466023 | C | T | 1.00 | 0.48 | 0.79 | 8.40E-05 |
| rs11627413 | 14 | 83802964 | G | C | 0.98 | 0.05 | 0.59 | 8.40E-05 |
| rs1969345 | 17 | 22064988 | G | A | 0.99 | 0.33 | 12.82 | 8.41E-05 |
| rs75025485 | 6 | 118201160 | A | G | 1.00 | 0.05 | 17.57 | 8.41E-05 |
| rs56806093 | 17 | 22062051 | C | CT | 0.99 | 0.33 | 1.28 | 8.46E-05 |
| rs6473355 | 8 | 83360125 | A | G | 0.99 | 0.31 | 0.77 | 8.46E-05 |
| rs368650727 | 13 | 95255336 | A | ATT | 0.91 | 0.30 | 12.78 | 8.47E-05 |
| rs7585610 | 2 | 3521051 | C | T | 1.00 | 0.16 | 13.77 | 8.48E-05 |
| rs35327823 | 13 | 109443648 | C | T | 1.00 | 0.08 | 0.65 | 8.49E-05 |
| rs1850917 | 17 | 22063186 | T | C | 0.99 | 0.33 | 12.82 | 8.50E-05 |
| rs60035268 | 4 | 1087487 | G | A | 0.98 | 0.13 | 14.19 | 8.53E-05 |
| rs4889756 | 17 | 22209193 | T | G | 0.99 | 0.35 | 12.80 | 8.54E-05 |
| rs137869374 | 22 | 38401362 | C | A | 0.99 | 0.02 | 22.63 | 8.55E-05 |
| rs1850920 | 17 | 22113123 | A | G | 1.00 | 0.34 | 12.82 | 8.55E-05 |
| rs4465156 | 1 | 183331860 | C | T | 1.00 | 0.12 | 0.69 | 8.56E-05 |
| rs36180633 | 7 | 155725299 | G | A | 0.99 | 0.16 | 13.80 | 8.59E-05 |
| rs147297381 | 1 | 150352869 | A | AT | 1.00 | 0.11 | 14.58 | 8.61E-05 |
| rs2900535 | 9 | 114721483 | G | A | 1.00 | 0.21 | 0.74 | 8.64E-05 |
| rs12866626 | 13 | 109425088 | A | G | 1.00 | 0.08 | 0.65 | 8.66E-05 |
| rs1887355 | 13 | 103235169 | T | C | 0.96 | 0.48 | 0.80 | 8.66E-05 |
| rs116812829 | 4 | 32735531 | T | A | 0.99 | 0.10 | 14.79 | 8.68E-05 |
| rs34682621 | 8 | 87677762 | TG | T | 1.00 | 0.12 | 0.70 | 8.70E-05 |
| rs72678007 | 4 | 127868973 | T | A | 0.99 | 0.05 | 17.03 | 8.81E-05 |
| rs1328152 | 9 | 73417351 | C | G | 0.98 | 0.20 | 13.33 | 8.83E-05 |
| rs78216697 | 10 | 54458178 | A | G | 0.99 | 0.11 | 0.69 | 8.85E-05 |
| rs12663983 | 6 | 40657007 | G | A | 1.00 | 0.27 | 13.00 | 8.87E-05 |
| rs13074515 | 3 | 28306022 | G | A | 1.00 | 0.17 | 13.73 | 8.87E-05 |
| rs146333707 | 2 | 16145619 | A | AT | 0.99 | 0.38 | 0.79 | 8.91E-05 |
| rs7406922 | 17 | 25475061 | T | G | 1.00 | 0.36 | 12.77 | 8.94E-05 |
| rs10772943 | 12 | 16674062 | A | G | 1.00 | 0.46 | 12.65 | 8.96E-05 |
| rs141634035 | 4 | 71771980 | A | G | 0.99 | 0.03 | 20.05 | 8.96E-05 |
| rs143354441 | 2 | 12769576 | T | A | 0.99 | 0.12 | 0.70 | 8.99E-05 |
| rs74444367 | 13 | 109424564 | G | A | 1.00 | 0.08 | 0.65 | 9.00E-05 |
| rs372838535 | 13 | 109424533 | CA | C | 1.00 | 0.08 | 0.65 | 9.02E-05 |
| rs9585940 | 13 | 103203070 | G | A | 1.00 | 0.37 | 12.75 | 9.02E-05 |
| rs10059836 | 5 | 25569869 | G | T | 1.00 | 0.14 | 0.71 | 9.02E-05 |
| rs61915305 | 12 | 16669088 | G | A | 1.00 | 0.39 | 12.73 | 9.03E-05 |
| rs201177649 | 6 | 152633085 | AT | A | 0.97 | 0.05 | 16.34 | 9.03E-05 |
| rs7662007 | 4 | 60396908 | C | T | 1.00 | 0.12 | 0.70 | 9.03E-05 |
| rs78941445 | 13 | 109424494 | A | G | 1.00 | 0.08 | 0.65 | 9.04E-05 |
| rs10610325 | 6 | 112548242 | TTTTA | T | 0.97 | 0.16 | 0.73 | 9.06E-05 |
| rs138387139 | 8 | 34980621 | TCTTGA | T | 0.97 | 0.13 | 13.97 | 9.07E-05 |
| rs78767631 | 13 | 109424427 | G | T | 1.00 | 0.08 | 0.65 | 9.08E-05 |
| rs8079074 | 17 | 25473921 | C | T | 1.00 | 0.36 | 1.28 | 9.12E-05 |
| rs41264469 | 1 | 150482255 | A | G | 0.99 | 0.12 | 14.31 | 9.13E-05 |
| rs1024844 | 12 | 16671432 | T | C | 1.00 | 0.46 | 12.64 | 9.14E-05 |
| rs11056989 | 12 | 16687223 | G | T | 0.99 | 0.32 | 12.86 | 9.18E-05 |
| rs34161875 | 13 | 109424116 | C | CT | 1.00 | 0.08 | 0.65 | 9.27E-05 |
| rs10930409 | 2 | 171100088 | G | A | 1.00 | 0.06 | 0.60 | 9.27E-05 |
| rs939730 | 17 | 25417579 | A | G | 1.00 | 0.36 | 12.76 | 9.29E-05 |
| rs12857877 | 13 | 109424041 | G | A | 1.00 | 0.08 | 0.65 | 9.32E-05 |
| rs9585937 | 13 | 103197489 | G | A | 1.00 | 0.37 | 12.74 | 9.32E-05 |
| rs192262825 | 4 | 57232423 | C | G | 0.99 | 0.02 | 0.42 | 9.32E-05 |
| rs17506553 | 13 | 103202770 | A | T | 1.00 | 0.37 | 12.74 | 9.32E-05 |
| rs59439912 | 8 | 24440678 | CAAA | C | 1.00 | 0.02 | 24.35 | 9.34E-05 |
| rs10190395 | 2 | 3516069 | A | T | 1.00 | 0.16 | 13.75 | 9.35E-05 |
| rs35943495 | 13 | 109423966 | C | T | 1.00 | 0.08 | 0.65 | 9.36E-05 |
| rs10204400 | 2 | 3503244 | C | T | 1.00 | 0.16 | 0.73 | 9.37E-05 |
| rs74647357 | 11 | 25169329 | G | T | 0.98 | 0.04 | 17.31 | 9.38E-05 |
| rs13010052 | 2 | 3524024 | C | T | 1.00 | 0.16 | 13.71 | 9.39E-05 |
| rs78184747 | 4 | 171387355 | G | A | 1.00 | 0.02 | 27.45 | 9.39E-05 |
| rs71435298 | 13 | 109423920 | G | A | 1.00 | 0.08 | 0.65 | 9.39E-05 |
| rs2441899 | 8 | 105332714 | A | G | 1.00 | 0.11 | 0.68 | 9.40E-05 |
| rs12293836 | 11 | 78711670 | T | C | 0.98 | 0.34 | 12.75 | 9.40E-05 |
| rs148120284 | 2 | 203224196 | T | C | 1.00 | 0.01 | 35.06 | 9.40E-05 |
| rs10204394 | 2 | 3503229 | C | T | 1.00 | 0.16 | 0.73 | 9.42E-05 |
| rs2077177 | 12 | 54418857 | T | C | 0.99 | 0.36 | 12.74 | 9.45E-05 |
| rs4657897 | 1 | 194000398 | T | C | 0.99 | 0.02 | 20.56 | 9.45E-05 |
| rs11056972 | 12 | 16669330 | G | A | 1.00 | 0.39 | 12.72 | 9.46E-05 |
| rs7309185 | 12 | 104512832 | G | A | 1.00 | 0.32 | 0.78 | 9.48E-05 |
| rs200165993 | 20 | 43369499 | CAA | C | 0.99 | 0.43 | 0.79 | 9.48E-05 |
| rs35875513 | 1 | 109530927 | GA | G | 0.93 | 0.28 | 12.76 | 9.49E-05 |
| rs9910055 | 17 | 42283037 | C | T | 1.00 | 0.27 | 13.01 | 9.49E-05 |
| rs10204409 | 2 | 3503269 | C | T | 1.00 | 0.16 | 0.73 | 9.52E-05 |
| rs41420550 | 5 | 91971171 | G | A | 0.99 | 0.04 | 17.67 | 9.54E-05 |
| rs17482617 | 13 | 109423624 | A | G | 1.00 | 0.08 | 0.65 | 9.58E-05 |
| rs1328153 | 9 | 73416062 | G | A | 1.00 | 0.20 | 13.39 | 9.60E-05 |
| rs3058584 | 8 | 25492386 | A | ACATTT | 0.98 | 0.05 | 0.60 | 9.60E-05 |
| rs71435296 | 13 | 109419473 | G | A | 1.00 | 0.08 | 0.65 | 9.62E-05 |
| rs10853131 | 17 | 25481292 | G | A | 1.00 | 0.36 | 12.77 | 9.67E-05 |
| rs17481980 | 13 | 109415914 | C | A | 1.00 | 0.08 | 0.65 | 9.68E-05 |
| rs17482022 | 13 | 109417175 | G | A | 1.00 | 0.08 | 0.65 | 9.68E-05 |
| rs71435295 | 13 | 109418440 | A | G | 1.00 | 0.08 | 0.65 | 9.68E-05 |
| rs34352192 | 13 | 109419719 | G | C | 1.00 | 0.08 | 0.65 | 9.68E-05 |
| rs71435297 | 13 | 109423457 | C | A | 1.00 | 0.08 | 0.65 | 9.69E-05 |
| rs936571 | 17 | 25480245 | A | G | 1.00 | 0.36 | 12.76 | 9.71E-05 |
| rs9298386 | 8 | 83364003 | C | G | 1.00 | 0.31 | 0.77 | 9.71E-05 |
| rs150791304 | 15 | 98505230 | T | A | 0.99 | 0.01 | 26.12 | 9.71E-05 |
| rs72933114 | 6 | 108056800 | C | T | 1.00 | 0.02 | 0.39 | 9.72E-05 |
| rs76487377 | 8 | 28241186 | C | T | 0.98 | 0.06 | 15.85 | 9.72E-05 |
| rs17391449 | 13 | 109423400 | G | A | 1.00 | 0.08 | 0.65 | 9.73E-05 |
| rs7159917 | 14 | 38562080 | A | G | 1.00 | 0.13 | 14.23 | 9.73E-05 |
| rs4739885 | 8 | 83363541 | C | G | 1.00 | 0.31 | 0.77 | 9.74E-05 |
| rs202139608 | 12 | 50111723 | C | CA | 1.00 | 0.02 | 0.44 | 9.75E-05 |
| rs77659082 | 6 | 118213400 | G | A | 1.00 | 0.05 | 17.45 | 9.76E-05 |
| rs58349034 | 6 | 166064529 | G | C | 1.00 | 0.01 | 0.34 | 9.77E-05 |
| rs1410373 | 9 | 73416797 | C | T | 1.00 | 0.20 | 13.38 | 9.78E-05 |
| rs7320528 | 13 | 103234450 | A | G | 0.95 | 0.36 | 12.62 | 9.79E-05 |
| rs7150667 | 14 | 23255897 | T | C | 0.97 | 0.06 | 0.64 | 9.79E-05 |
| rs72696859 | 1 | 150369895 | A | G | 1.00 | 0.11 | 14.60 | 9.80E-05 |
| rs3030797 | 5 | 1380021 | TAC | T | 0.98 | 0.15 | 1.37 | 9.81E-05 |
| rs1701470 | 10 | 12154833 | C | T | 1.00 | 0.30 | 12.92 | 9.82E-05 |
| rs2313740 | 17 | 25506858 | C | T | 1.00 | 0.36 | 12.75 | 9.83E-05 |
| rs11056991 | 12 | 16689618 | G | T | 0.99 | 0.30 | 0.78 | 9.84E-05 |
| rs9481732 | 6 | 118124789 | T | C | 1.00 | 0.05 | 0.57 | 9.85E-05 |
| rs12206874 | 6 | 111383559 | A | G | 0.98 | 0.33 | 1.27 | 9.85E-05 |
| rs79701990 | 12 | 50137122 | A | G | 1.00 | 0.02 | 0.40 | 9.86E-05 |
| rs4739884 | 8 | 83362039 | A | G | 1.00 | 0.31 | 0.77 | 9.87E-05 |
| rs7150679 | 14 | 23255914 | T | G | 0.97 | 0.06 | 0.64 | 9.87E-05 |
| rs17089687 | 4 | 60403334 | C | T | 1.00 | 0.13 | 0.71 | 9.87E-05 |
| rs10441548 | 8 | 83376488 | G | T | 1.00 | 0.31 | 0.77 | 9.88E-05 |
| rs56005245 | 8 | 128113426 | C | T | 1.00 | 0.23 | 13.24 | 9.89E-05 |
| rs35592421 | 3 | 168361229 | T | C | 1.00 | 0.32 | 0.78 | 9.89E-05 |
| rs79555073 | 13 | 109417870 | G | A | 1.00 | 0.08 | 0.65 | 9.89E-05 |
| rs148474018 | 2 | 16134188 | TTC | T | 0.91 | 0.30 | 0.79 | 9.91E-05 |
| rs11988084 | 8 | 83361359 | C | T | 1.00 | 0.31 | 0.77 | 9.92E-05 |
| rs1555299 | 20 | 43370106 | T | C | 1.00 | 0.42 | 0.79 | 9.92E-05 |
| rs4595506 | 11 | 16542089 | A | G | 1.00 | 0.47 | 12.63 | 9.95E-05 |
| rs67214773 | 5 | 113455496 | C | G | 1.00 | 0.36 | 0.78 | 9.97E-05 |
| rs10929405 | 2 | 16135423 | C | T | 0.99 | 0.46 | 0.79 | 9.97E-05 |
| rs35377895 | 8 | 83360905 | GT | G | 1.00 | 0.31 | 0.77 | 9.98E-05 |
| rs12971629 | 19 | 20502708 | G | A | 0.97 | 0.24 | 0.77 | 9.98E-05 |
| rs117382201 | 14 | 33072781 | G | A | 0.99 | 0.03 | 0.47 | 9.99E-05 |
| rs10055075 | 5 | 25570843 | A | T | 1.00 | 0.14 | 0.71 | 1.00E-04 |
